# FOXA1 mutations co-opt nascent transcription factor networks in partnership with androgen receptor to enhance prostate tumorigenicity

**DOI:** 10.1016/j.celrep.2026.116950

**Published:** 2026-01-31

**Authors:** Erik M. Ladewig, Abbas Nazir, Tyler Park, Vinson B. Fan, Zhendong Cao, Jacob Hawk, Lauren Kelly, Robert Tjian, Christina S. Leslie, Charles L. Sawyers

**Affiliations:** 1 Computational and Systems Biology Program, Memorial Sloan Kettering Cancer Center, New York, NY 10065, USA; 2 Human Oncology and Pathogenesis Program, Memorial Sloan Kettering Cancer Center, New York, NY 10065, USA; 3 Department of Molecular & Cell Biology, University of California Berkeley, Berkeley, CA 94720, USA; 4 These authors contributed equally; 5 Lead contact

## Abstract

Mutations in the pioneer transcription factor FOXA1 occur in 10%–40% of prostate cancers and broadly alter chromatin accessibility. In a cohort of 874 primary and metastatic tumors, we confirm frequent Wing2 missense mutations and indels, as well as C-terminal truncating frameshifts. To define their functional impact, we performed single-nucleus multiome profiling in mouse prostate organoids expressing representative alleles, including overexpressed wild-type FOXA1. Each subgroup produces distinct chromatin and transcriptional changes, but all perturb epithelial lineage specification. Indel mutants promote basal-like states, whereas C-terminal truncations, Wing2 missense mutations, and elevated wild-type FOXA1 drive secretory L1-like luminal fates. Integrated RNA-seq, ATAC-seq, and ChIP-seq reveal that L1-like specification involves a hybrid androgen receptor/FOXA1 motif and cooperation with POU2F1. *In vivo*, these same alleles, combined with Trp53/Pten loss, shift tumor histology from basal-like to secretory luminal phenotypes.

## In brief

Ladewig et al. show that distinct Foxa1 mutations reprogram chromatin, altering epithelial lineage specification. Indels promote basal-like states, whereas C-terminal truncations, Wing2 missense mutations, and FOXA1 overexpression drive L1 luminal differentiation via an AR-FOXA1 hybrid motif and POU2F1 cooperation. *In vivo* modeling recapitulates basal-like to L1 phenotype with Trp53/Pten loss.

## Graphical Abstract

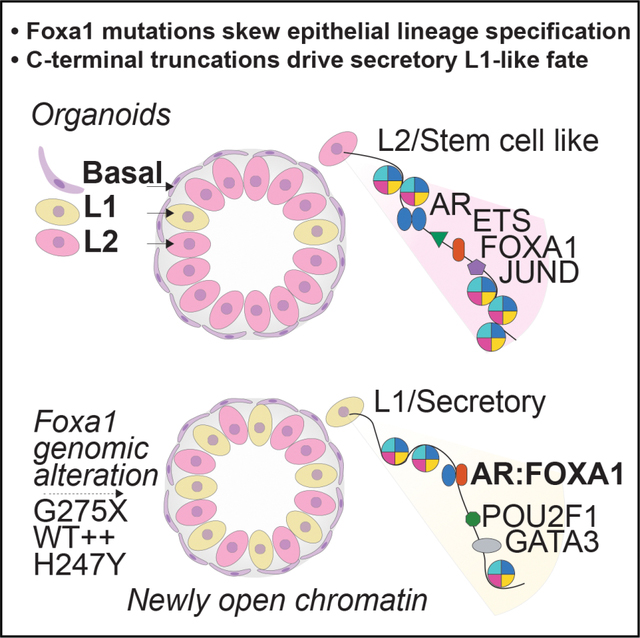

## INTRODUCTION

FOXA1 encodes a developmentally critical pioneer transcription factor (TF) that becomes an oncogenic driver in human prostate and breast cancer through distinct types of genomic alterations.^1–3^ FOXA1 mutations are present in ~12% of prostate cancer patients in Western cohorts and ~40% in Asian cohorts. Curiously, this ethnicity difference is also seen with ERG translocations but in the reverse direction, common in Western cohorts but rarer in Asian cohorts. Notably, FOXA1 and ERG alterations are mutually exclusive across ethnicities and collectively account for ~60% of all prostate cancer worldwide.

The most frequent FOXA1 alterations are missense or indel mutations in the Wing2 region of the Forkhead domain.^1,4^ Nonsense mutations that lead to C-terminal truncation define a third group, which is reported to be exclusive to metastatic disease.^2,5^ A fourth group of FOXA1 alterations is defined by elevated levels of WT FOXA1 mRNA and protein due to focal gene amplification, FOXA1 promoter or 5′ UTR mutations, or translocations.

Several groups have explored the biological consequences of mutant FOXA1 expression in cell lines, organoids, and mice. Mutant FOXA1 expression results in widespread chromatin accessibility changes enriched for canonical and non-canonical Foxa1 binding motifs within days of induction, suggesting sequence-specific DNA binding of mutant FOXA1 is a key first step for aberrant pioneering.^1^ FOXA1 confers mutant-specific effects on prostate epithelial cell fate, ranging from activation of basal-like to pro-luminal gene expression programs depending on the mutation, as well as tumorigenicity in mice.^1,2,5,6^

In an expanded FOXA1-mutant prostate cancer cohort (*n* = 874), we confirm the high prevalence of missense, indel (in frame), and frameshift mutations with improved resolution, compared to earlier reports.^1,2,4^ Notably nearly all indels localize to either M253 or F254 (*n* = 335), highlighting the functional importance of these residues in Wing2. We also identify truncating (nonsense) mutations occurring at similar frequencies in primary cancer and metastatic tumors, differing from earlier reports.^2,5^

For mechanistic characterization, we performed transcriptional and TF motif accessibility analyses at single-cell resolution (scRNA+ATAC-seq) to examine underlying cell state transitions. This integrated single-cell approach revealed that all FOXA1 mutants impact prostate epithelial lineage fate. The most consistent and prominent effects were rapid increases in the percentage of basal cells (by expression of mutants at M253 and E255, representing the two indels) or L1-like secretory luminal cells (by expression of mutants with C-terminal truncation, H247 missense or excess WT). The appearance of L1 cells in organoid culture is remarkable because, although L1 is the predominant cell type in mouse and human prostate glands *in vivo*, they are poorly represented in organoid culture, due to growth conditions that favor stem-like (L2) cells.

Through integrated RNA-seq, ATAC-seq, and ChIP-seq analysis, we identified a non-canonical composite FOXA1-AR binding motif and the homeodomain POU2F1 TF as uniquely associated with and required for induction of L1 luminal fate. Direct comparison of the truncation mutant (G275X) with WT revealed that absence of the C terminus shortened the time required for cells to reach the L1 state (24 h vs. 5 days), as well as chromatin residence time in single-molecule tracking (SMT) experiments, consistent with a “fast” mechanism postulated earlier.^2^ These mutants also induce an L1-like state *in vivo*, initiating a phenotypic switch to secretory luminal histology in a background of *Pten*/*Trp53* loss, a widely used model of prostate cancer with predominantly basal-like features. Our analyses therefore uncovered a TF network comprising FOXA1-AR and POU2F1 that drives the L1 luminal secretory fate and enhances tumorigenicity downstream of mutant FOXA1 expression.

## RESULTS

### Updated map of FOXA1 mutants in human prostate cancer

**Figure 1.**
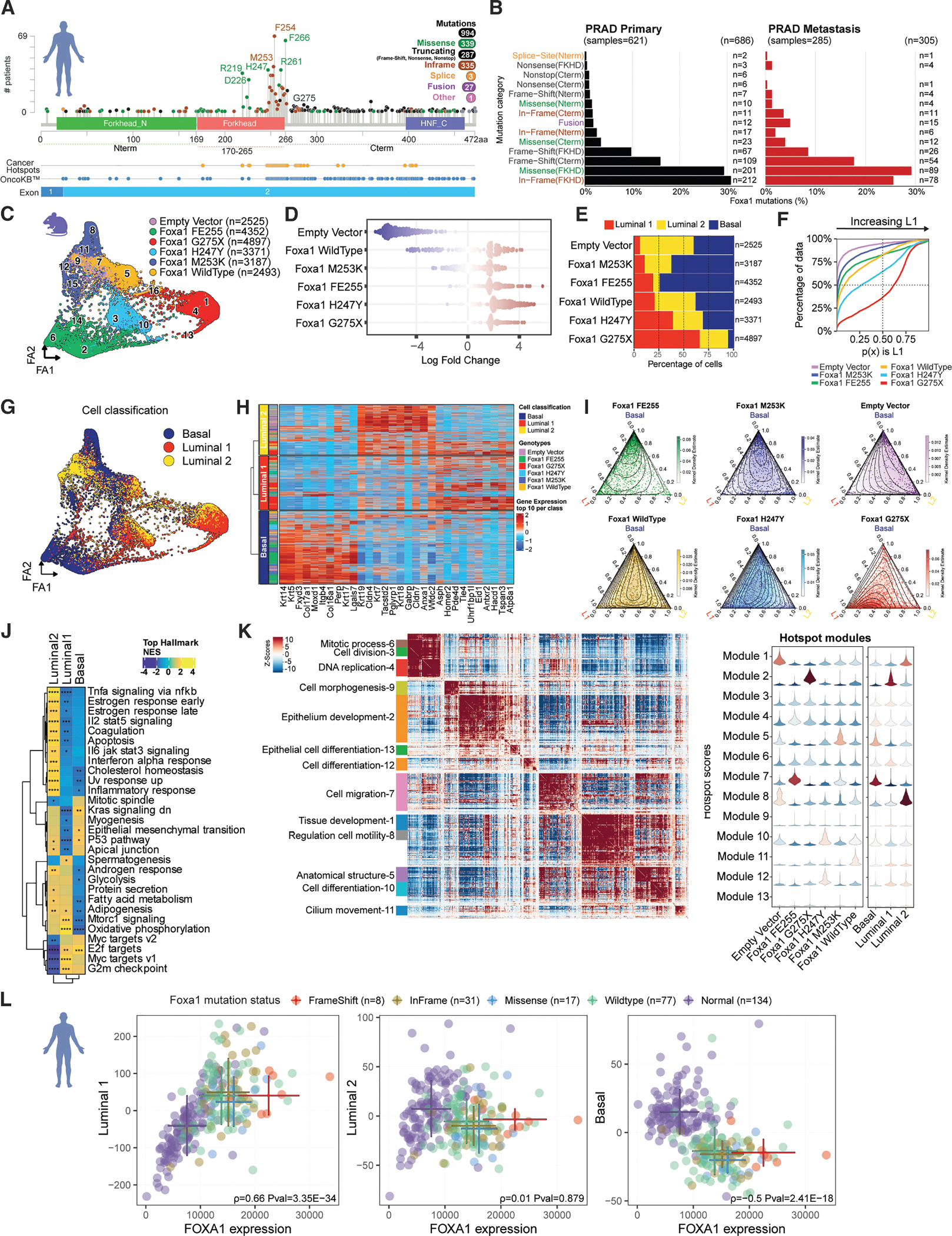
FOXA1 mutations drive epithelial cell-specific transcriptomes in prostate organoids (A) A lollipop plot of the human *FOXA1* gene, its protein domains, and the location and number of mutations seen in prostate cancer cohorts obtained through cbioportal.org (see STAR Methods). Frequent mutations are labeled. (B) Bar plots organized by mutation type in primary and metastatic prostate adenocarcinoma (PRAD), least to most frequent (top, bottom). (C) Force-directed layout of scRNA-seq mouse prostate organoids colored by genotype, with Leiden clusters labeled. (D) Cell neighborhood analysis graph produced by Milo showing neighborhood log fold changes by genotype.^8^ (E) Bar plot of cell type proportions per genotype. (F) ECDF of L1 cell probabilities per genotype. (G) Force-directed layout of scRNA-seq colored by cell type. (H) Heatmap of most significantly differential genes in L1, L2, and basal. (I) Ternary plots showing basal/L1/L2 probability distributions with density contours. (J) GSEA categories shown on heatmap ranked by normalized enrichment scores indicating enrichment in L2, L1, and basal cells. (K) Hotspot gene modules with GO terms (left) and module scores by genotype (right). (L) Scatterplots of human prostate cancer (PCa) samples stratified by *Foxa1* mutation: frameshift (red), in-frame (orange), missense (blue), wildtype (green), and normal (purple). Categories (except normal) derive from PCa samples; “wildtype” refers to FOXA1-intact. Error bars show mean ± standard deviation.

Prior work mapping the location of FOXA1 mutations in human prostate cancer relied on cohorts with ~89–370 independent FOXA1-mutant cancers, including primary tumors and metastases.^1,2,4^ Leveraging the paired germline and tumor sequencing routinely performed at Memorial Sloan Kettering Cancer (MSK) using the MSK-IMPACT test,^7^ we identified 686 primary prostate cancers and 305 metastatic prostate cancers with somatic FOXA1 mutations (Figures 1A and 1B; Table S1; STAR Methods). Consistent with earlier analyses of smaller cohorts, mutations fall into three subgroups: missense (*n* = 339), indels (inframe; *n* = 335), and truncation (nonsense; *n* = 287). As expected, missense mutants and indels, which collectively account for 68% of FOXA1 mutations in this cohort, map almost exclusively to Wing2 in the Forkhead domain, whereas truncation mutations (29% of FOXA1 mutations) are spread across the C terminus distal to Fkhd. Of note, truncation mutants are present with equal frequency in primary tumors and metastases, a finding that differs from another cohort.^2,5^ Another striking finding is nearly all indels map to just two residues within Wing2 (M253, E255). A cohort of this size linked to clinical outcome data provided an opportunity to explore prognostic differences across mutation subgroups. Toward that end, patients with tumors with indels (inframe mutations at M253 or E255) have improved survival compared to those with truncating (nonsense) mutations (Figure S1A).

### Oncogenic FOXA1 mutants promote a spectrum of luminal and basal lineage fates

Building on our earlier work documenting gain of function in chromatin opening activity of FOXA1 mutations, we selected a representative Wing2 missense mutation (H247Y), the two indel mutations (M253K, FE255), a truncation mutation just downstream of the Forkhead domain (G275X), and WT for deeper investigation into how these chromatin accessibility changes impact transcription and oncogenesis. Because of their presumed role in disease initiation, we examined their activity in freshly isolated primary mouse prostate cells grown in organoid culture, a system that we and others have optimized to study dynamic events in prostate cancer initiation and progression *in vitro* and *in vivo* following orthotopic transplantation.^9–11^ Robust expression of Flag-tagged WT and of each mutant allele was confirmed at levels well above endogenous FOXA1, albeit with WT somewhat lower than each of the mutant subclasses (Figures S1B and S1C).

We next used single-cell multiome sequencing to simultaneously profile transcriptome and chromatin accessibility differences in comparison to an empty vector (EV) control. We clustered cells by their single-cell transcriptomes and visualized them using a 2D force-directed layout (with ForceAtlas [FA]; Figure 1C). Cells clustered primarily by mutant, with 12/16 (75%) clusters containing 75% or more cells from a single genotype (Figures S1E and S1F). Only two clusters contained cells from all samples: cluster 14, enriched in cell cycle genes, and cluster 16, dominated by ribosomal RNA (Figures S1D and S1G). We further performed neighborhood-based differential abundance tests for cells of each mutant genotype vs. EV using MILO (see STAR Methods). The computed log fold changes over neighborhoods show that cells expressing FE255, H247Y, and G275X diverged the most from EV control (Figure 1D).

To examine mutant-specific clustering, we noted that the most statistically significant differentially expressed genes (DEGs) that distinguish the FOXA1 clusters included luminal (*Krt8* and *Krt19*) and basal cell (*Krt5*, *Krt14*, and *Krt15*) marker genes, suggesting that different FOXA1 mutations bias cells to distinct epithelial fates (Figure S1H). Prostate tissue contains two major epithelial cell types: (1) basal cells, which line the basement membrane, and (2) luminal cells, which secrete the protein constituents of seminal fluid. The luminal cell compartment includes two primary subtypes: (1) differentiated secretory cells (called L1 or distal Lum), which account for >90% of luminal cells in the prostates of hormonally intact males and (2) stem-like cells (called L2 or proximal Lum; 5%–10% of luminal cells).^12,13^ Importantly, the ratio of L1 to L2 cells *in vivo* is reversed in organoid culture, where L1 cells are poorly maintained due to growth conditions that favor growth of basal and L2 luminal cells.^14^ Indeed, we found that L1 cells (annotated using literature-derived signature for L1, L2, and basal cells) account for only 6% of epithelial cells in EV cultures. Conversely, cultures expressing each of the five mutant alleles (including elevated WT) had increased levels of L1 cells, ranging from 10% (M253K), 19% (FE255), 20% (WT), 39% (H247K), and 66% (G275X; Figure 1E). These findings were supported by a Gaussian mixture model, using the ECDF of the L1 posterior probability (Figures 1F and S1I–S1K; STAR Methods) and further validated by morphological evidence of large cystic lumens in organoid bright-field images (Figure S1L), consistent with enhanced secretory activity. UMAP visualization revealed cell type clusters largely driven by epithelial lineage phenotypes, suggesting FOXA1 mutant transcriptomes generate variation among defined cell types (Figure 1G). Differential expression analysis of basal, luminal 1 (L1), and luminal 2 (L2) cell types identified basal (*Krt5* and *Krt14*) and luminal genes (*Krt18* and *Krt19*) and L1- (*Tspan3* and *ATp8a1*) and L2-specific (*Cldn4* and *Tacstd2*) genes (Figure 1H; Table S2; STAR Methods). Ternary plots further quantified these identities, where WT, H247Y, and G275X cells clustered preferentially toward the L1 vertex (Figure 1I). While it is possible the expanded proportion of L1 cells is due to faster proliferation, the magnitude and speed with which this occurs (11-fold over 5 days for G275X), coupled with the amount of chromatin remodeling, supports a lineage conversion model.

Although all FOXA1 mutants increased in L1 cells, the FE255 and M253K alleles also led to a significant increase in basal cells (Figure 1E), consistent with prior independent findings for the F254E255 mutant.^6^ Thus, a primary consequence of oncogenic FOXA1 expression is a shift in epithelial lineage specification, with indel mutants (M253K and FE255) showing basal skewing, whereas truncation (G275X), missense (H247Y), and WT all display secretory L1-like skewing.

To move beyond lineage specification, we performed Gene Set Enrichment Analysis (GSEA) and found upregulated biological pathways such as oxidative phosphorylation, glycolysis, androgen response, and mTORC1 signaling in the L1 cell clusters from G275X and H247Y (clusters 4 and 10, respectively), as well as MYC and E2F targets in all clusters of FE255 and G275X (Figures 1J, 1K, and S1M). To identify biologically meaningful modules across Foxa1 mutants, we applied Hotspot analysis,^15^ yielding 13 modules shared across all mutants that are associated with oncogenesis, including mitotic processes (module 6), cell division (module 3), and DNA replication (module 4; Figure 1K, left panel). Other programs such as epithelium development (module 2) and cell migration (module 7) were linked to specific mutants (G275X and FE255, respectively). Interestingly, these mutant-specific modules are similarly enriched in the epithelial lineage associated with each of those mutants (L1 cells and basal cells, respectively; Figure 1K, right panel).

To contextualize our murine findings within human disease, we analyzed data from the Chinese Prostate Genome and Epigenome Atlas,^4^ a cohort with sufficient numbers of FOXA1 mutant cases (56 FOXA1-mutant tumors from an RNA-seq cohort of 134 tumor/normal pairs; see STAR Methods) with matched tumor/normal expression profiles and annotated FOXA1 mutation status. We evaluated FOXA1 expression and L1, L2, and basal epithelial gene signatures across distinct mutation categories (Figure 1L; Table S3; STAR Methods). Except for normal tissue, all categories represent prostate cancer (PCa); thus, “Wildtype” refers to FOXA1-intact PCa. L1 activity positively correlated with *FOXA1* expression in normal and in tumor samples. Notably, tumors with FOXA1 frameshift mutations showed the highest FOXA1 expression as well as L1 activation. L2 signature scores correlated with *FOXA1* in normal tissues but not in tumors. Basal signature activity was inversely associated with FOXA1 expression, which was generally reduced in tumor tissue. Unlike mouse organoids, patients with inframe mutants did not have evidence of basal lineage skewing, albeit with a limited number of cases.

In summary, all subclasses of FOXA1 mutants represented by these alleles activate transcriptomic changes that skew the relative proportion of basal, progenitor-like (L2) luminal and differentiated/secretory (L1) luminal cells in primary organoid culture, together with induction of mitotic programs associated with oncogenesis. Notably, the L1 skewing profile matches transcriptomic features in FOXA1-mutant human cancers.

### Chromatin opening initiated by mutant FOXA1 alleles reveals lineage-specific TF motifs

**Figure 2.**
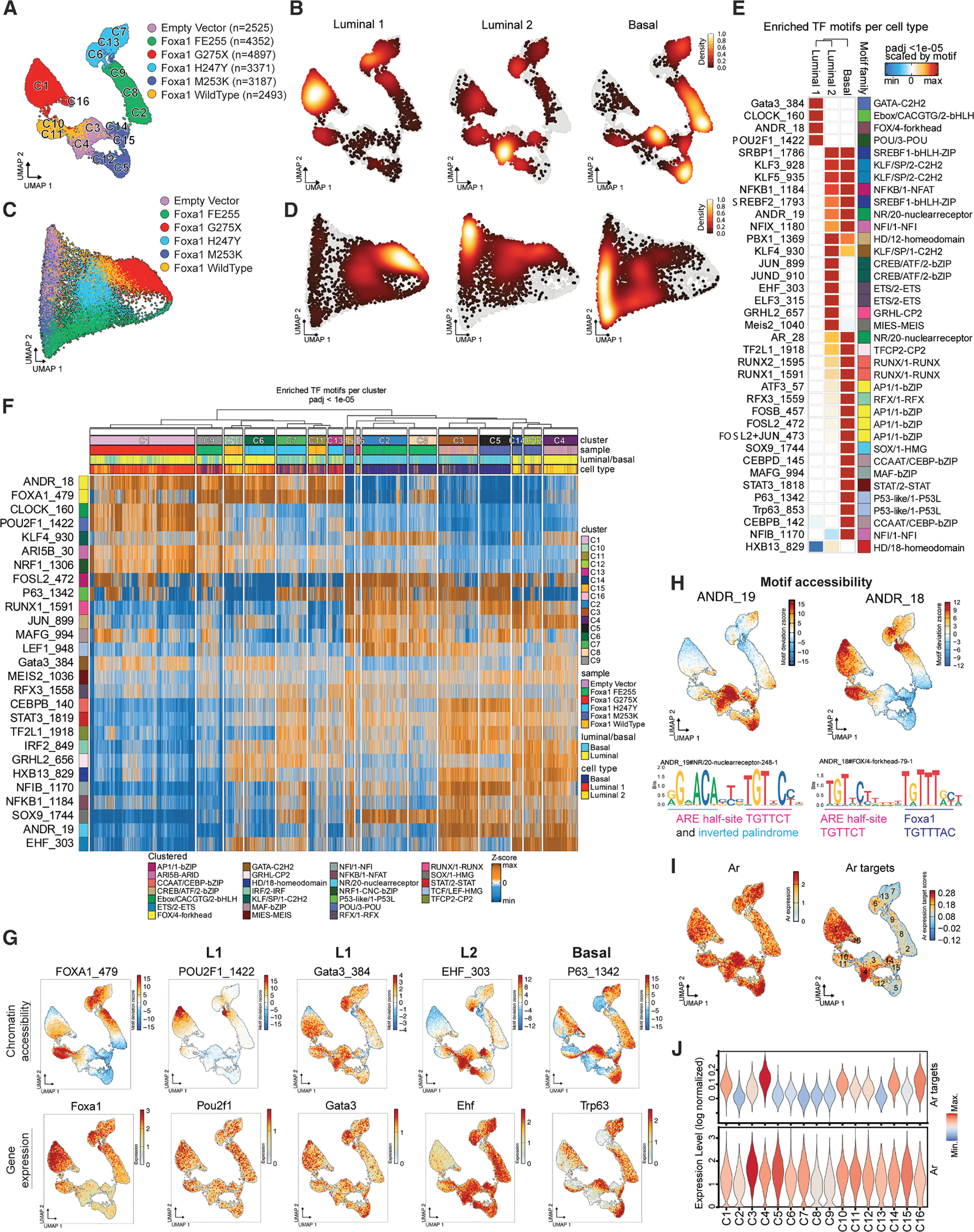
FOXA1 single-cell chromatin accessibility reveals distinct transcription factor motifs that integrate luminal/basal expression programs (A) scATAC-seq uniform manifold approximation and projection (UMAP) of mouse prostate organoids. (B) UMAPs show density of cells per epithelial subtype. (C) scATAC-seq force-directed layout projected from alternative CellSpace embedding. (D) CellSpace-generated force-directed layout shows density per epithelial subtype. (E) Heatmap of significant motifs, also labeled by motif family, assessed by epithelial subtype and colored according to −log10 (adjusted *p* value). (F) Average accessibility for top significantly accessible and expression correlated transcription factor (TF) motifs. (G) Individual UMAPs of chromatin accessibility (top) and gene expression (bottom) for several key TF motifs. (H) UMAPs for AR canonical motif ANDR_19 and composite motif ANDR_18 accessibility assessed by chromVAR (top left and top right, respectively). The ANDR_19 motif sequence with bracketed androgen response element (ARE) half-site and inverted palindromic ARE site (bottom left). ANDR_18 hybrid motif of ARE half-site and Foxa1 (bottom right). (I) scATAC-seq UMAPs showing *Ar* expression, indicating broad expression in genotypes (left). scATAC-seq UMAPs showing high AR target gene expression, concentrated in regions containing mostly L1 and L2 cell types (right). (J) Violin plots of *Ar* target expression (top) and *Ar* expression (bottom).

We next analyzed chromatin accessibility induced by each **FOXA1** allele. Cells were subjected to quality filters (Figure S2A), represented by variable genomic tiles, clustered using a graph-based approach, and visualized by Uniform Manifold Approximation and Projection (UMAP) (Figures 2A and S2B). This representation primarily clustered cells by genotype, revealing distinct chromatin accessibility profiles among mutants, consistent with the large number of mutant-specific accessible peaks detected in the scATAC-seq data (Figure S2C). The FOXA1 G275X mutant exhibited the highest number of differential peaks relative to the EV control, and the largest cluster (C1) overlapped with a dense region of L1 cells (Figures 2B, S2D, and S2E). The presence of coordinated accessibility and transcriptional shifts across nearly all cells within each genotype suggests that **FOXA1** pioneering activity operates uniformly within individual cultures.

The discovery that FOXA1 mutants with similar transcriptomic programs (e.g., L1 luminal with G275X, H247Y, and WT) exhibit distinct ATAC profiles led us to investigate whether TF motifs in newly accessible regions drive FOXA1-dependent expression changes. Using the sequence-informed scATAC-seq embedding algorithm CellSpace,^16^ which maps DNA k-mers and cells into a shared latent space, we uncovered striking chromatin state overlaps among mutants. These states, organized into basal, L1 luminal, and L2 luminal clusters anchored at the three corners of the embedding, reveal that while each FOXA1 mutant exhibits unique chromatin accessibility, shared lineage-specific TF binding signals underlie the three canonical epithelial states (Figures 2C and 2D).

We next identified key TF motifs with chromatin accessibility linked to TF expression within each epithelial state (STAR Methods). Basal-like cells showed open chromatin with TRP63 (a basal cell marker) and SOX9 motifs (Figures 2E–2G). Ar+ basal and L2 cells (C3, C4, C5, and C14) showed enrichment of the canonical AR motif ANDR_19, consisting of an inverted palindromic repeat followed by the androgen receptor element (ARE) half-site (AGAACA…TGTTCT). L2 cells also displayed motifs for ELF3 and EHF (ETS family TFs), paralleling luminal progenitor cells in breast tissue,^17^ along with HOXB13 (an AR cofactor and regulator of AR transcriptional activity).^18,19^

Surprisingly, in L1 cells, the ANDR_19 motif was inaccessible despite strong AR target gene activation (Figure 2H). Instead, these cells showed enrichment of an alternative hybrid Ar:Foxa1 motif (ANDR_18: TGTTCT…TGTTTG) (Figures 2E and 2F). L1 cells (G275X cluster) exhibited the highest AR target gene expression, aligned with the accessibility of ANDR_18 (Figure 2H). In contrast, basal clusters (C3, C5; EV, M253K genotypes) enriched for ANDR_19 had lower AR target expression despite higher Ar mRNA levels (Figure 2J).

As suggested from CellSpace analysis, we also noted lineage-specific enrichment of other TF motifs, including POU2F1/OCT1, involved in the regulation of development,^20^ and GATA3, expressed in breast and prostate luminal cells (Figures 2E–2G). Despite evidence implicating *Gata2* in AR-dependent prostate cancer,^21,22^ we did not find evidence of *Gata 2* expression in mouse organoid models (Figure S2F). In summary, lineage-specific enrichment of ANDR_18 vs. ANDR_19 motifs, coupled with different magnitudes of AR target gene activation (despite comparable levels of Ar expression), suggests that FOXA1 perturbs the AR cistrome to dictate distinct epithelial cell states in cooperation with additional partner TFs.

### FOXA1/AR colocalization at composite binding motifs is associated with lineage-specific TF expression

**Figure 3.**
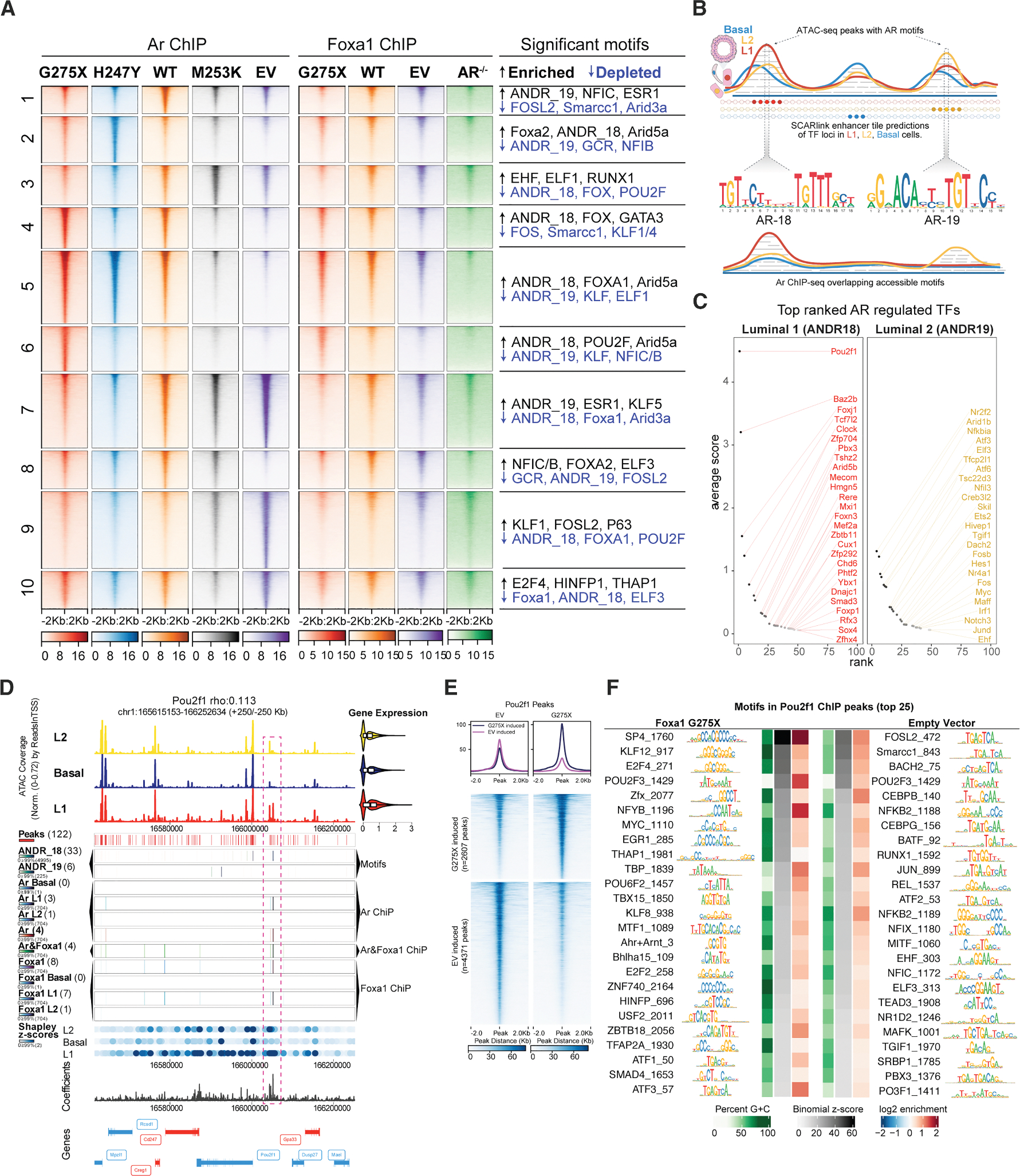
FOXA1 G275X cooperates with the androgen receptor by enriching for an alternative Ar motif and reinforces Ar target gene expression (A) Tornado plots of AR ChIP-seq (left) or FOXA1 ChIP-seq (right) data separated by AR-driven clustering. Significantly accessible motifs detected in AR ChIP-seq data per cluster are shown on the far-right side and indicated as more binding (enriched) or less binding (depleted). (B) Schematic of scATAC-seq, ChIP-seq, and SCARlink analysis to nominate genes with Ar-regulated enhancers. scRNA-seq gene expression is estimated from scATAC-seq for each cell type (L1, L2, and basal). scATAC-seq is tiled into 500-nt bins using SCARlink around gene bodies (+−250 kb), where each tile is scored using Shapley. Significant Shapley scores designate candidate enhancers per gene that are overlapped with accessible AR motifs (ANDR18 and ANDR19) and AR ChIP-seq peaks. The combined scores are averaged and used to rank potential Ar-regulated transcription factors (TFs). (C) Average SCARlink scores per TF motif that contain the ANDR_18 motif in AR ChIP peaks within all luminal 1 regions (left panel) or basal regions (right panel) (STAR Methods). (D) *Pou2f1* genomic locus ( ±250 kb) with tracks listed top to bottom: (1) normalized chromatin accessibility for scATAC-seq in L2, basal, and L1; (2) significant peak detection above background (adjusted *p* value < 0.05); (3) motif detection for ANDR_18; (4) ANDR_19; and (5) Foxa1. Bulk AR ChIP-seq tracks separated into (6) basal, (7) L1, (8) L2, and (9) total AR; 10) intersection of peaks in both AR and FOXA1; 11) ChIP-seq for total FOXA1; FOXA1 separated into (12) basal, (13) L1, and (14) L2. Shapley values from SCARlink loci separated into (15) L2, basal, and L1, then (16) SCARlink coefficients track, and finally (17) gene annotations track. (E) Tornado plots showing significantly differential peaks (adjusted *p* value < 0.05 and log2 FC > 0.5) in empty vector (EV; left) vs. FOXA1 G275X (right). (F) POU2F1 ChIP motif enrichment in G275X and EV mouse prostate organoids (left and right, respectively). The 25 most highly significant motifs (ranked by binomial *Z* score) are ordered by significance, from greatest (top) to least (bottom).

Having identified a hybrid AR:FOXA1 motif linked to enhanced AR output and L1 luminal cell fate, we conducted chromatin immunoprecipitation sequencing (ChIP-seq) experiments to search for biochemical evidence of AR-FOXA1 cooperativity, focusing on FOXA1 mutants that promoted high L1 cell content (G275X, H247Y, and WT), with M253K and EV serving as controls. Analysis of 67,811 AR ChIP-seq peaks grouped into ten clusters revealed that motifs enriched in L1 cells from scATAC-seq data (Figure 2E) were also detected in Ar ChIP-seq (Figures S3A and S3B). These include the composite hybrid motif ANDR_18, along with POU2F1 and GATA3 transcription factor (TF) motifs (clusters 2, 4, 5, and 6; Figure 3A). Interestingly, these same motifs were enriched in FOXA1 ChIP-seq data from G275X and WT organoids, supporting co-binding by AR and FOXA1. In contrast, the canonical AR motif ANDR_19 was absent from these peaks but significantly enriched in EV and M253K organoids (cluster 7), where ANDR_18 was depleted. This divergence suggests distinct motif preferences between L1-promoting FOXA1 mutants and non-L1 states. To explore whether usage of ANDR_18 or ANDR_19 motifs is associated with differential response to AR inhibition, we performed proliferation assays in the presence or absence of enzalutamide. G275X- and WT-expressing organoids (both with ANDR_18 usage) were more sensitive to AR inhibition than EV or FE255 (ANDR_19; Figure S3F), consistent with the greater castration sensitivity of L1 cells in the normal prostate *in vivo.*^12^ However, the sensitivity of H247Y organoids (also ANDR_18 and pro-L1) was indistinguishable from EV, suggesting L1-like signatures are not always linked with AR dependence. Indeed, a subset of L1 cells in the normal mouse prostate persist following castration.^12^

The selective enrichment of POU2F1 and GATA3 motifs in WT, H247Y, and G275X organoids suggested that these TFs act as downstream targets of the hybrid ANDR_18 motif. To test this hypothesis, we conducted a genome-wide SCARlink analysis, integrating scATAC-seq and scRNA-seq data to identify lineage-specific enhancers in L1, L2, and basal states. Enhancers were segmented into 500-nt tiles and ranked using SCARlink-derived Shapley scores, then cross-referenced with Ar ChIP-seq peaks and accessible AR motifs (ANDR_18 or ANDR_19) to pinpoint Ar-regulated enhancers (Figure 3B^23^; STAR Methods). This analysis identified POU2F1 as the top AR-regulated TF candidate in L1 cells associated with the ANDR_18 composite motif (Figures 3C and 3D, left). Similarly, investigation of the ANDR_19 motif in L2 cells identified three ETS family genes (*Elf3*, *Ets2*, and *Ehf*) as potential AR-regulated targets (Figure 3C, right). ATAC-seq data confirmed selective enrichment of ELF3 and EHF motifs in L2 cells (Figure 2E), highlighting their relevance to L2-specific gene regulation.

Further analysis underscored the potentially pivotal role of POU2F1 in L1 cells. For example, the GATA3 motif, prominently enriched in L1 cells (Figure 2E), was also present in L2 cells (Figure 2F), aligning with its established function in luminal cell identity in breast and prostate tissues.^24^ However, POU2F1 motif enrichment was exclusive to L1 cells (Figures 2E–2G and 3D). Consistent with motif analysis, POU2F1 ChIP-seq revealed changes in the genomic distribution of POU2F1 in G275X cells compared to EV, as well as enrichment for other TF motifs, including KLF (KLF12 and KLF8), E2F (E2F4 and E2F2), and MYC (Figures 3E and 3F). FOXA1 and POU2F1 motifs were also highly enriched in a bulk ATAC-seq study of 26 primary human prostate adenocarcinomas (100 and 84 percentiles, respectively).^25^ Together, these findings point to POU2F1 as a central AR-regulated TF in defining L1 identity.

### POU2F1 is a critical downstream effector of the pro-luminal (L1) phenotype

**Figure 4.**
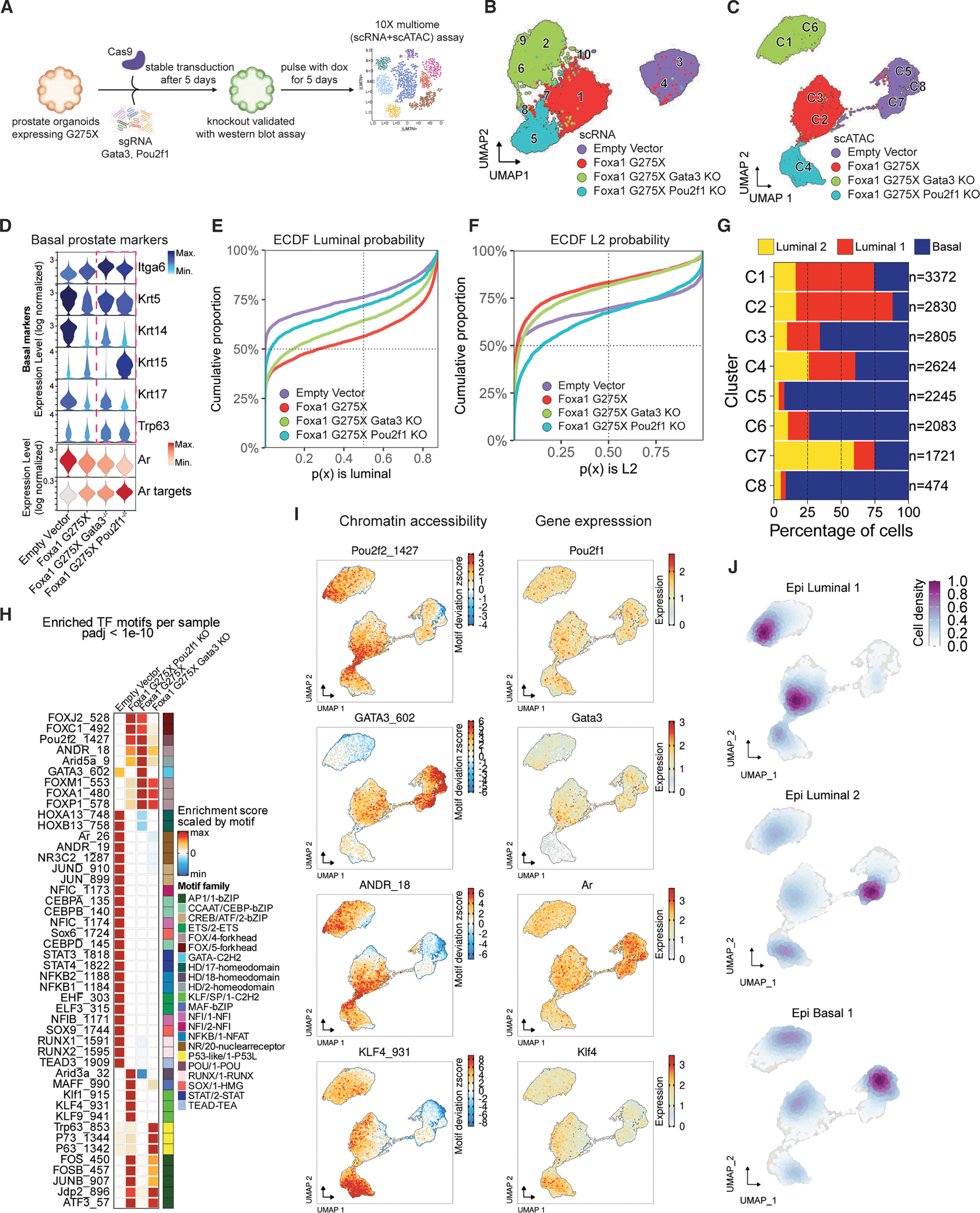
FOXA1 G275X promotes POU2F1 and GATA3 transcription factor activity to maintain luminal identity (A) Experimental schematic for control and G275X organoids with CRISPR knockout of *Pou2f1* or *Gata3*. After Cas9/gRNA transduction and KO validation, organoids were doxycycline-induced for 5 days and profiled by 10x multiome. (B) scRNA-seq UMAP of the 4 genotypes: EV, G275X, G275X *Gata3−/−*, and G275X *Pou2f1−/−*. (C) scATAC-seq UMAP of multiome samples. (D) Violin plots show increased basal marker expression in knockouts with reduced *Ar* expression and elevated AR-target expression. (E) ECDF of luminal probabilities in EV, G275X, and G275X knockout cells. Lower lines indicate higher luminal probabilities, i.e., most to least probable luminal cells: G275X, G275X *Gata3* KO, G275X *Pou2f1* KO, and EV. (F) ECDF of L2 probabilities ordered most to least probable: G275X *Pou2f1* KO, EV, G275X *Gata3* KO, and G275X. (G) Cell percentage per cluster bar plot. (H) Heatmap of the chromVAR motif accessibility across genotypes. Motifs shown with adjusted *p* value < 1×10^−10^. (I) ChromVAR accessibility UMAPs (left) and gene expression (right) for POU2F1/2, GATA3, ANDR_18, and KLF4. (J) UMAPs indicating density of cells for L1, L2, and basal-like cells. L2 increase in G275X *Pou2f1* KO cells compared to G275X and G275X *Gata3* KO.

Having implicated *Pou2f1* as a direct AR-FOXA1 target gene, we used CRISPR targeting to assess if POU2F1 is required for induction of the L1 transcriptional program. We selected G275X for these studies based on robust induction of the L1 signature (Figures 1E and 1F). Although not directly implicated as an AR-FOXA1 target by SCARlink (Figure 3C), we included *Gata3* because its motif was selectively enriched in L1 cells (Figure 2E). After confirming CRISPR deletion of each TF at the protein level (Figure S4A), we performed single-cell multiome (scRNA+scATAC) sequencing 5 days after Dox induction (Figure 4A).

As expected from our initial analysis (Figures 1A and 2A), UMAP projections of the scRNA-seq as well as the scATAC-seq data revealed clear separation between EV and G275X (Figures 4B and 4C). CRISPR deletion of either *Pou2f1* or *Gata3* resulted in substantial shifts of the parental G275X RNA-seq and ATAC-seq clusters, implicating both TFs in shaping the G275X-driven transcriptome. Curiously, *Gata3* mRNA levels were nearly absent in G275X *Pou2f1* KO cells, suggesting POU2F1 regulates *Gata3* expression, consistent with the presence of two POU2F1-binding peaks at the *Gata3* locus in our ChIP-seq data (Figure S4H). To explore the gene programs responsible for these UMAP shifts, we noted increased expression of basal-associated genes (*Itga6*, *Krt5*, and *Trp63*) following *Pou2f1* or *Gata3* KO, as well as striking upregulation of *Krt15* following *Pou2f1* deletion (Figure 4D). Signature analysis using ECDF plots confirmed that G275X increases the probability of L1 fate specification (Figures 4E and 4F). This signal was mitigated by deletion of either *Gata3* or *Pou2f1*. Moreover, in the case of *Pou2f1* deletion, the reduction in L1 fate specification was replaced by a shift toward the L2 fate (Figures 4D–4F), also seen in the chromatin accessibility data (Figure S4I).

We next examined the consequences of *Pou2f1* and *Gata3* deletion on the motifs specifically enriched in G275X organoids. *Pou2f1−/−* cells lost accessibility of the FOXA1 motif, implicating POU2F1 as a key TF responsible for sustaining the FOXA1 cistrome in L1 cells (Figures 4H, 4I, and S4J). We observed an increase in motif accessibility for KLF4, a Kruppel family and Yamanaka factor, in G275X cells following *Pou2f1* deletion, noteworthy because KLF motifs are selectively enriched in L2 cells (Figure 4I). GATA3 accessibility was lost in *Gata3* KO cells, as expected but also in *Pou2f1* KO cells likely due to loss of *Gata3* mRNA expression. However, the changes in *Foxa1* and ANDR_18 seen following *Pou2f1* deletion were not seen in *Gata3−/−* cells (Figure 4H). *Gata3* deletion also resulted in gain of accessibility for the basal TRP63 motif, consistent with the established role of GATA3 in luminal fate specification. Taken together, our results implicate POU2F1 as a key effector of L1 luminal identity, based on loss of FOXA1 and ANDR_18 motifs and gain of L2-associated KLF family TF motifs upon *Pou2f1* deletion. POU2F1 appears to operate upstream of *Gata3*, but the role of GATA3 in lineage specification appears to be pan-luminal, as evidenced by increased accessibility of basal TF motifs (TRP63) and loss of L1 and L2 transcriptomes following *Gata3* deletion.

### G275X has a shorter chromatin residence time and induces luminal identity faster than WT

A previous study of mutant FOXA1 alleles found that Wing 2 mutants have shorter chromatin residence times compared to WT based on fluorescence recovery after photobleaching (FRAP) and single-particle tracking (SPT) assays.^2^ One caveat of this work was the use of overexpressed FOXA1 alleles, which can lead to non-physiologic effects, particularly with TFs. To determine if the G275X mutant also has a shorter chromatin dwell time, we used Cas9-mediated genome editing to insert HaloTag into the endogenous FOXA1 locus of LNCaP-AR prostate cancer cells and then derived several clones expressing either FOXA1 WT-Halo or G275X-Halo (Figure S5C). SPT trajectories were extracted and subjected to nonparametric Bayesian inference^26^ to estimate the proportion of molecules bound to chromatin vs. freely diffusing throughout the nucleoplasm. We found that substantially less G275X is associated with chromatin than WT. Similarly, G275X recovered significantly quicker than WT in FRAP experiments (Figures S5A and S5B). Consistent with earlier work on other Wing 2 mutants, these results show that G275X has a shorter dwell time than WT (a so-called “fast” mutant).^2^

**Figure 5.**
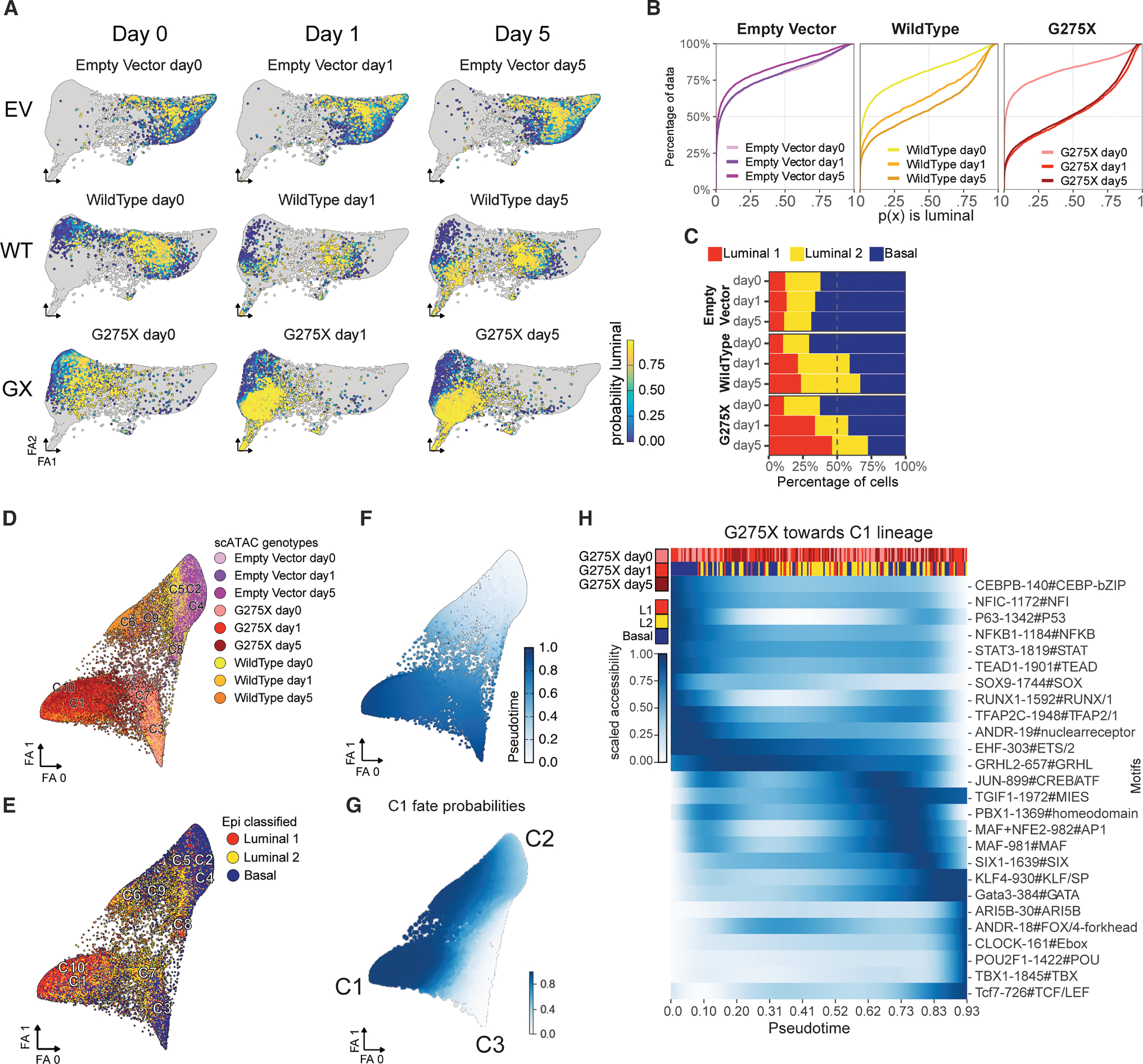
G275X mutant leads to faster activation of L1 luminal identity (A) UMAPs shown by day and genotype, with each cell colored by luminal probability. (B) ECDF curves for day 0, 1, and 5 showing stable low luminal probability in EV, with early increases in WT and G275X. (C) Bar plot of epithelial cell type by day and genotype showing L1 increase in WT and G275X starting at day 1, with greater increase in G275X. (D) scATAC-seq UMAP of cells colored by genotype. (E) scATAC-seq UMAP of cells colored as basal, L2, and L1. (F) scATAC-seq UMAP colored by Palantir pseudotime, from early (top) to two terminal endpoints (bottom left/right). (G) Fate probabilities as a function of pseudotime toward two branches (C3 and C1). (H) Heatmap of smoothed motif accessibility ordered by pseudotime. Top row shows G275X time points (day 0→1→5), and second row shows basal/L2/L1 annotations; darker blue indicates higher accessibility.

To explore whether the “fast” phenotype might be linked to the L1 lineage specification phenotype, we performed a time-course experiment to ask whether the L1 signature is activated more quickly following G275X s. WT Foxa1 induction by comparing scMultiome profiles at 24 h vs. 5 days. Luminal cell probabilities determined by ECDF of scRNA-seq data remained stable in EV organoids throughout the time course but progressively increased from day 1 to day 5 in WT organoids, as expected. In contrast, maximal luminal probability (~75% L1+L2) was reached after 24 h in G275X organoids (Figures 5A–5C). The change in scATAC-seq profile of G275X organoids, visualized by UMAP, was similarly fast, reaching its “destination” endpoint within 24 h, whereas the scATAC-seq profile of WT organoids evolved over 5 days (Figures 5A and S5G). Commensurate with our earlier TF motif analysis, the composite FOXA1:AR (ANDR_18) and POU2F1 motifs linked to L1 fate specification were accessible within 24 h in G275X organoids but to a lesser extent in WT (Figure S5H). Thus, shorter chromatin residence time of G275X relative to WT, measured by SPT and FRAP, correlates with more rapid chromatin accessibility and transcriptomic changes. This result is consistent with growing evidence that TF residence time measured by SPT is often short (seconds), followed by a transcriptional burst seconds later, whereas the sequence of downstream transcriptional changes required for lineage specification takes much longer.^27,28^

To investigate the sequence of TF activities required to establish luminal identity, we performed pseudotime analysis across the organoid time series (Figure 5F). This revealed a predominance of basal cells at day 0 (cluster C2, defined primarily by EV cells), followed by bifurcation into two branches representing luminal (L1) cells (cluster C1, primarily G275X and WT cells at days 1 and 5) and a basal-like cluster (C3 seen in WT and G275X; Figure 5G). Next, we calculated and plotted smoothed pseudotime estimates for the TF motifs enriched in L1 cell fate (ANDR_18, POU2F1, GATA3, and ARID5B; Figure 5H). The results show that ANDR_18 and GATA3 motifs appear early in the L1 fate transition, coincident with loss of TRP63 motifs. POU2F1 appears later, suggesting it contributes to the specification of L1 cells from L2 cells. Later in pseudotime, GATA3 motif accessibility increases further, consistent with our transcriptomic data suggesting that Pou2f1 functions upstream of GATA3 (Pou2f1 knockout results in loss of *Gata3* expression). Taken together, pseudotime analysis in concert with time-series multiome profiling predicts sequential steps in TF activation that orchestrate the transition toward the L1 fate.

### Pro-L1 FOXA1 mutants drive luminal identity *in vivo*

The capability of FOXA1 mutants to drive secretory luminal cell lineage programs typically associated with terminal differentiation raises intriguing questions about their oncogenic potential, given that epithelial cancers often exhibit stem-like characteristics and impaired differentiation. To explore this unusual association, we examined the three FOXA1 alleles that induce an L1 luminal phenotype (WT, H247Y, and G275X) in tumorigenicity assays.

**Figure 6.**
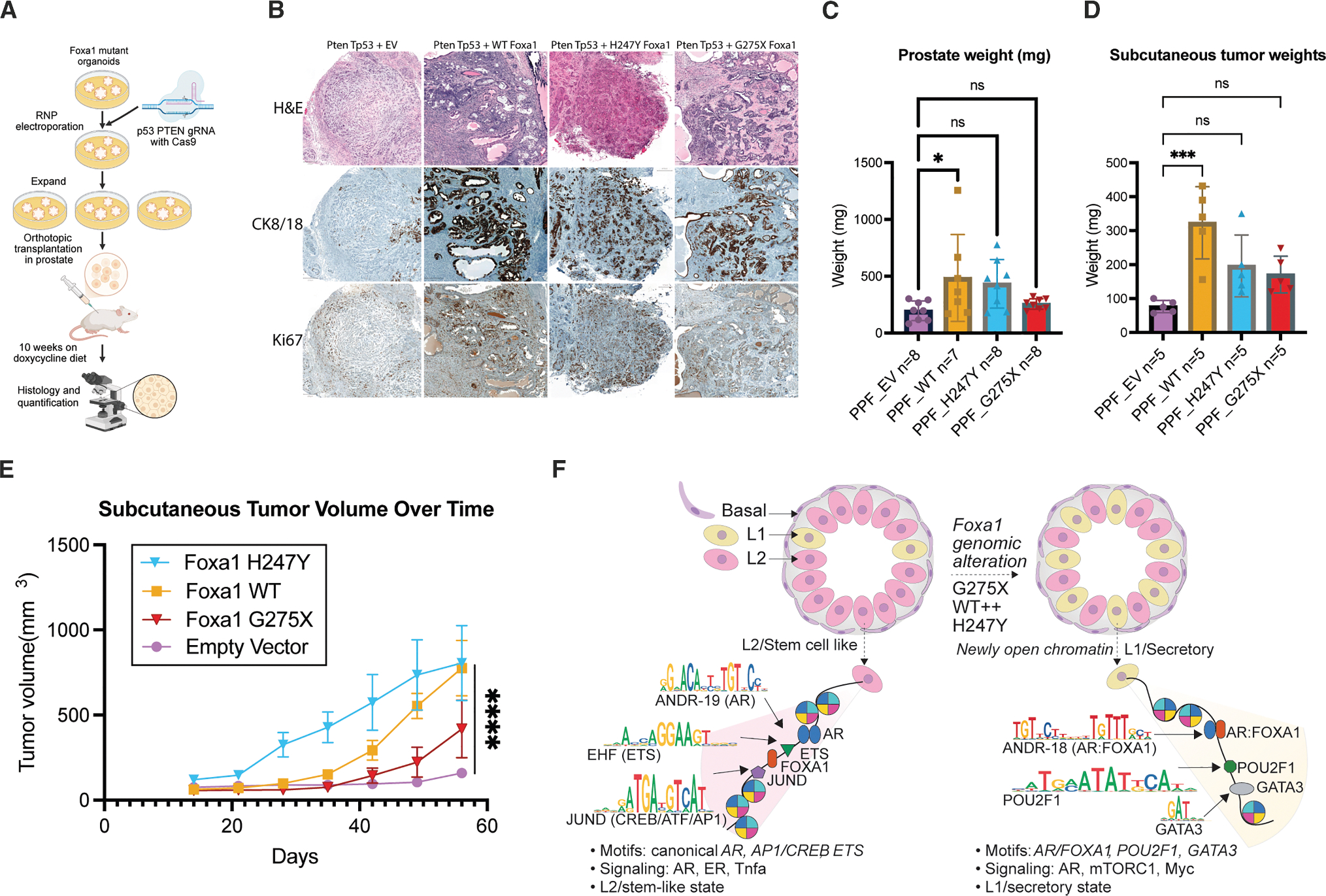
Wing2 mutants boost tumorigenicity while maintaining luminal identity (A) Experimental schematic of Foxa1 mutant, *Pten*/*Trp53* KO organoid generation. These organoids were used for *in vivo* transplantation, subcutaneous in the right flank and orthotopic inside the prostate. The mice were maintained on a doxycycline diet for the 10-week duration of the *in vivo* experiment. (B) Histology from orthotopic tumors of mice injected with mutant Foxa1-expressing organoids (WT, H247Y, and G275X) display robust Ck8/18+ expression, mirroring the luminal adenocarcinoma phenotype seen in FOXA1-mutant human cancers. (C) Prostate weight comparisons of mutant genotypes: WT, H247Y, and G275X compared to EV. One-way ANOVA used to determine significance between groups, followed by Dunnett’s test for multiple comparisons. (D) Subcutaneous tumor weight comparisons of mutant genotypes: WT, H247Y, and G275X compared to EV. One way ANOVA test used to determine significance between groups, followed by Dunnett’s test for multiple comparisons. (E) Tumor growth curves for all genotypes (H247Y, WT, G275X, and EV); interaction term significant by two-way ANOVA (*p* < 0.0001). (F) Model: FOXA1-mutant organoids (WT overexpression, H247Y, and G275X) show increasing L1 vs. L2 cell proportions. L1 cells exhibit open chromatin with AR:FOXA1 composite, POU2F1, and GATA3 motifs and upregulated AR/mTORC1 signaling. L2 cells display canonical AR, AP1, and ETS motifs with upregulated ER/TNFα signaling.

We previously demonstrated that several FOXA1 mutations promote tumorigenesis in a subcutaneous model but only in the context of *Pten* loss.^1^ Here, we expanded this observation but now using prostate organoids with combined *Pten*/*Trp53* deletion, a background that generates basal-like (Ck5+) tumors upon orthotopic transplantation, allowing us to evaluate the luminal-inducing effects of FOXA1 mutants *in vivo* (Figure 6A).

In mice injected with EV organoids (*Pten*^−/−^; *Trp53*^−/−^), invasive tumors with poorly differentiated, basal-like (Ck5+; Ck8/18−) features developed within 10 weeks. In contrast, organoids expressing either WT, H247Y, or G275X FoxA1 produced tumors with moderate-to-well differentiated histology and strong Ck8/18+ expression, closely resembling luminal adenocarcinomas observed in FOXA1-mutant human cancers (Figure 6B). The tumors with WT or H247Y FOXA1 also had increased proliferation (Ki67) and resulted in greater prostate weights (Figure 6C). Similar findings were observed in subcutaneous implantation models, where all three FOXA1 alleles accelerated tumor growth while driving luminal differentiation (Figures 6D and 6E). In summary, FOXA1 mutations that specify L1 luminal differentiation *in vitro* display similar activity *in vivo*, converting the basal-like histology seen with *Pten*/*Trp53* loss alone to a canonical luminal histology resembling that seen in FOXA1-mutant human prostate cancer.

## DISCUSSION

The high prevalence of FOXA1-mutant prostate cancer, particularly among Asian men, underscores the need for deeper understanding of the pathobiology and clinical behavior of these tumors. Through analysis of 874 such cases (2–3 times larger than previous cohorts), we confirm previously reported mutation patterns that clearly segregate into 3 main subgroups: missense (Wing2), indel/inframe (also Wing2), and nonsense (truncation). A fourth subgroup includes tumors with elevated levels of WT FOXA1 due to gene amplification or alterations that are common but more challenging to identify through panel-based sequencing. With increased sample size, several new insights emerged: truncation mutations occur at similar frequencies in primary and metastatic tumors, and indels cluster strongly at two residues (M253, F254), accounting for 31% of all indels. Access to linked clinical data also suggests truncation mutations may be associated with shorter survival, though this requires independent validation.

To explore the pathobiology across these 4 subclasses, we employed integrative single-cell analysis using freshly derived mouse prostate epithelial cells. Mutant FOXA1 reshapes the chromatin landscape via sequence-specific binding at canonical motifs. Missense mutations (H247Y), truncations (G275X), and elevated WT (modeling FOXA1 gene amplification) redirected AR to previously inaccessible FOXA1-AR hybrid motifs (ANDR18) and activated transcriptional programs that promote L1 luminal differentiation, mirroring features seen in human prostate cancers with analogous FOXA1 mutations. Specifically, G275X co-opts POU2F1/OCT1 as a master regulator, establishing aberrant transcriptional circuits within days of FOXA1 expression (Figure 6F). Conversely, indel mutations (M253 and FE255) activated basal-like transcriptional programs, although we were unable to confirm basal skewing in human tumors, likely due to small sample size (*n* = 17). Notably, prostates in mice engineered to express a different indel mutation (R265–71) have L1-like features,^5^ indicative of intraclass heterogeneity depending on the specific mutation. Another variable in both studies is the reliance on mouse models to explore the transcriptomic consequences of mutant FOXA1 expression. Our decision to model disease initiation in primary prostate epithelial cells rather than in prostate cancer cell lines that contain other oncogenic drivers was intentional. As technologies for culturing primary human prostate epithelial cells improve, analogous studies in human models will be essential.

The discovery of the FOXA1-AR hybrid motif (ANDR18) suggests a mechanism for how FOXA1 reprograms AR activity and helps reconcile earlier conflicting reports of FOXA1 as both an activator and inhibitor of AR signaling.^29,30,31^ Such discrepancies likely reflect AR cistrome repositioning from the canonical ANDR19 motif to hybrid motifs like ANDR18. ANDR18-enriched AR enhancers were also a prominent feature in the L1-like tumors that developed in the FOXA1 R265–71 mouse model mentioned above,^5^ as well as in a recent study of NSD2 in AR/FOXA1-dependent enhancers.^32^ Further investigation is warranted to determine whether lineage changes induced by the FOXA1/AR hybrid motif depend on altered AR activity.

Our integration approach, combining single-cell multiomics, ChIP-seq, time course analyses, and computational tools such as SCARlink, enabled rapid identification of cooperating TF circuitry, which we validated through functional studies. The identification of POU2F1/OCT1 as a key effector of mutant FOXA1-driven L1 luminal lineage specification serves as an instructive example. Building on earlier work implicating POU2F1/OCT1 and other TFs in AR signaling,^21^ our single-cell strategy now defines the precise TF circuitry underlying L1 lineage fate specification. Mutant FOXA1 establishes an aberrant AR/FOXA1 cistrome (through binding to a hybrid FOXA1-AR motif), with subsequent POU2F1/OCT1 activation. Future studies may investigate other highly ranked FOXA1-cooperative TFs, such as CLOCK, ARID5B, and KLF4, through approaches like Perturb-seq.

Whereas missense and indel mutations all map to the Wing2 domain, nonsense/truncation mutations span the entire C terminus downstream of the Forkhead domain, suggesting greater heterogeneity within this subclass. Indeed, the pronounced L1 luminal phenotype induced by G275X contrasts with the stem-like (L2) phenotype produced by another C-terminal truncation (P358fs), emphasizing the need for a broader allelic series to better understand this subclass. This complexity gains more clinical relevance given the preliminary association between truncations and shorter patient survival relative to missense mutations.

Interestingly, the pro-L1 luminal differentiation program driven by many of these oncogenic FOXA1 mutations contrasts with the stem-like programs typically associated with epithelial malignancies. Similar luminal differentiation programs are activated in ERG-translocated prostate cancers^33^ and SPOP-mutant tumors, which show elevated PSA expression and enhanced sensitivity to anti-AR pathway therapy.^34,35^ Together with ERG and SPOP, which collectively account for 70%–80% of prostate cancers, a substantial fraction of FOXA1 mutants share a unifying theme of activating pro-luminal fate specification programs coupled with oncogenicity.

### Limitations of the study

Because FOXA1 mutations likely act as cancer-initiating events, it is important to study their effects in primary (normal) prostate cells lacking additional mutations. We therefore used primary mouse prostate organoids rather than human prostate cancer cell lines, most of which harbor genomic alterations (e.g., ETS translocations) often mutually exclusive with FOXA1 mutations. Currently, mouse organoids provide a more tractable platform for these types of experiments than human models, but our conclusions may be limited by interspecies differences. Our study confirmed that C-terminal truncation mutants constitute a distinct, albeit heterogeneous, FOXA1 mutant subtype, with mutations spanning the entire C terminus that are equally prevalent in primary and metastatic disease. However, the lineage specification phenotype within this subtype may be more complex than revealed here through characterization of the single G275X truncation and will require investigation through a larger allelic series. Similarly, although C-terminal truncations were associated with a worse prognosis, this observation does not account for possible intragroup heterogeneity. Larger cohorts with linked clinical outcomes will be necessary to refine these clinicogenomic associations.

### RESOURCE AVAILABILITY

#### Lead contact

Further information and requests for resources and reagents should be directed to and will be fulfilled by the lead contact, Charles Sawyers (sawyersc@mskcc.org).

#### Materials availability

All data are available in the main text or supplemental information.

#### Data and code availability

- The raw and processed RNA-seq and ATAC-seq datasets generated in this study have been deposited in GEO and made publicly available under accession number GSE285574, also listed in the key resources table.
- No original code is associated with this study.
- Any additional information needed to reanalyze the data is available upon request from the lead contact.

## STAR★METHODS

### EXPERIMENTAL MODEL AND STUDY PARTICIPANT DETAILS

#### Animals

All animals (mice) used in the study were cared for in accordance with guidelines approved by the Memorial Sloan Kettering Cancer Center (MSKCC) Institutional Animal Care and Use Committee (IACUC) and Research Animal Resource Center (RARC). For orthotopic transplants, NOD SCID Gamma (NSG) mice were purchased from Jackson laboratories, and 1 million cells were injected into the dorsal lobe of the prostate in 8-week-old mice. Only male mice were used in this study. Cells were prepared after dissociating 3D organoids into single cell suspension. For each mouse, the appropriate number of cells were resuspended in 10ul of matrigel and 10ul of organoid media, a total volume of 20ul. For subcutaneous injections, organoids were dissociated, and 1 million cells were resuspended in matrigel and media, in 1:1 ratio. 20ul of cell suspension was then implanted in the right flank of a NOD SCID Gamma (NSG) mice were purchased from Jackson laboratories.

#### Cell lines

Cell lines used in this study were maintained in a 37 °C and 5% CO2 incubator. Cell lines were not authenticated but periodically tested and found negative for mycoplasma test (Lonza #LT07–318). LNCaP/AR cells were derived within the lab.

#### Organoids

Organoids were periodically tested and found negative for mycoplasma test (Lonza #LT07–318). Murine organoids were established from adult male mouse prostate taken from C57/BL6 mouse, as previously described.^12^ Briefly, entire mouse prostate was harvested, and physically dissociated into smaller pieces, with sterile razor blades. Next, it was digested with collagenase type II (Gibco) for about 1hr at 37 °C with gentle shaking. Next, TrypLE (Gibco) was added for digestion at 37 °C until a single-cell suspension was obtained. Single cell suspension was washed 3 times with cold PBS after TrypLE digestion. Organoid media was prepared as previously described.^12^ It was freshly supplemented with Y-27632 (10uM) to inhibit anoikis. Finally, cells were filtered through FACS tubes and seeded for organoid culture. Bulk isolated prostatic epithelial cells were embedded in 35-μL drops of basement membrane extract (Matrigel, Corning) and overlaid with mouse prostate organoid medium.

For transduction, organoids were transduced with lentivirus for pCW-EV and pCW-Foxa1 (WT and mutant cDNA) and were selected with 2ug/ml of puromycin for 5 days. The pCW plasmids were cloned as previously described using Flag-tagged alleles.^1^ Antibiotic selection was started 3–4 days after viral transduction. Organoid sorting and 3D culturing was performed as described previously.^1^ Transduction with Lenti-Cas9-Blast was followed by 5-day of selection in 10ug/ml of blasticidin. For most *in vitro* assays assessing the effect of the Foxa1 transgene, unless otherwise mentioned, organoids were seeded and treated with doxycycline at 500ng/ml to induce the expression of the Foxa1–2A-dsRED fusion cDNA and were harvested 5 days after the addition of doxycycline.

#### Human cohort of FOXA1-mutant prostate cancer

Prostate cancer data containing **FOXA1** mutations were obtained from cbioportal.mskcc.org. After filtering for relevant cases and mutation types, the dataset comprised **991 mutations** (primary = 686; metastasis = 305) across **906 cases** from **874 patients**. The filtered dataset is provided in Table S1.

#### Analysis of human CPGEA cohort data

Normalized RNA-seq data from the CPGEA cohort was obtained and analyzed for mutation categories and expression of FOXA1. Table S3 contains mutation and Gene expression scores.

To assess epithelial versus FOXA1 expression in FOXA1-mutant tumors we analyzed processed RNA-seq data from the Chinese Prostate Genome and Epigenome Atlas (*n* = 134 cases with matched tumor/normal profiles and annotated FOXA1 mutation status).^4^ Epithelial output scores were computed using gene signatures derived from normal mouse prostate.^12^ These mouse signatures were converted to human orthologs, and expression values were standardized as z-scores across all samples. Briefly, z-scores were calculated by subtracting the gene’s mean expression across all samples from each individual sample and dividing by the standard deviation of gene expression across all samples. For each sample, the sum of z-scores for L1, L2, or basal genes yielded an epithelial output score. FOXA1 expression was then plotted against each epithelial score in separate panels (Figure 1L).

### METHOD DETAILS

#### LnCAP-AR cell culture and endogenous Foxa1-HaloTag knock-in

LnCAP-AR cells^37^ were cultured with RPMI-1640 with 9.1% FBS with penicillin and streptomycin and kept in a water-jacketed incubator at 37°C with 5% CO2. Cells were generally split 1:8 every three days with 0.25% Trypsin to facilitate detachment.

HaloTag was knocked-in at the endogenous FOXA1 locus with plasmid-encoded SpyCas9, sgRNAs, and homology directed repair templates. For WT Foxa1, the sgRNA targeted the C terminus of the protein. For Foxa1-truncation, the sgRNA targeted the ORF where the truncation occurs. In both cases, repair templates containing a linker and HaloTag were cloned with ~500 bp upstream and downstream homology to the cut site. The following Cas9 protospacer-PAM sequences were used for endogenous HaloTag editing:

WT-guide: CGGGTCTGGaatacacacct[tgg].

GX-guide: caagtgcgagaagcAGCCGG[GGG].

LnCAP-AR cells were electroporated with all plasmids above using a homemade buffer^38^ and the T-009 protocol of the Lonza Nucleofector II. Single clones were isolated by FACS and sequence-validated to have the by PCR and Sanger sequencing. Clones used for this study were also Western blotted for Foxa1. The cell lines tested negative for mycoplasma about monthly by PCR and were confirmed to be LnCAP cells by STR profiling.

#### Lentivirus production and transduction

Human HEK293 cells (ATCC. CRL-1573) were grown in DMEM (ATCC. 30–2003) with 10% FBS, 100 U/mL penicillin-streptomycin and 2mM L-glutamine. Lentivirus packaging was performed in HEK293T using Lipofectamine. 2000 reagent (Invitrogen) in accordance with the manufacturer’s instructions. Medium containing virus was concentrated using PEG-it Virus Precipitation Solution (System Biosciences). Lentiviral transduction was performed as described previously.^12^

#### Organoid western blots

Organoids were isolated from the matrigel basement membrane extract by typle E (trypsin) treatment and performing multiple washes with ice-cold PBS in a 15-mL Falcon tube. Cells were lysed in RIPA buffer containing protease inhibitors (Calbiochem) and phosphatase inhibitors (Calbiochem) on ice and sonicated three times for 30 s at 30-s intervals using a Bioruptor. Lysate were centrifuged for 10 min at 20,000G at 4deg. The supernatant was collected as protein lysate. Protein concentrations were quantified using a bicinchoninic acid (BCA) assay (Pierce, Thermo Fisher). Lysates were denatured using 4X protein loading dye (SDS 200 nM Tris, 8% SDS, 0.4% bromophenol blue, 40% glycerol, 400 mM 2-mercaptoethanol, pH 6.8). 10–20 μg of protein was loaded on NuPage 4–12% gradient bis-tris polyacrylamide gels (Invitrogen). After electrophoresis, protein was transferred to a PVDF membrane and blocked with either 5% milk or 5% BSA in TBS-T. Primary antibodies were incubated overnight. Membranes were washed using TBS-T and incubated with secondary antibodies for 1 h at room temperature. Proteins were visualized using ECL and ECL prime (Amersham, GE healthcare) and ImageQuant 800 (Amersham, GE healthcare). Blots were analyzed using ImageJ software. The following antibodies were used in the assay, FOXA1 sigma, SAB2100835–100UL (1–1000 dilution), Vinculin cst, 13901S (1–5000 dilution), Pou2f1 abcam, ab15112 (1–1000 dilution), Gata3 abcam, ab199428 (1–1000 dilution), Cyclophillin B cst, 43603S (1–5000 dilution), and TBP abcam, ab63766 (1–5000 dilution).

#### Immunohistochemistry (IHC) and immunofluorescence (IF)

H and E staining and IHC was carried out by the MSKCC Molecular Cytology Core using Ventana Roche Benchmark Ultra and Leica. For tissue fixation, murine tissue was fixed using 4% paraformaldehyde overnight, dehydrated with ethanol, and paraffin embedded per standard protocol. 4-μm slides were cut and placed on glass slides. Immunohistochemistry or immunofluorescence of FFPE tissue was performed using a Ventana BenchMark Ultra and Leica. FFPE stained tissue was scanned using a MIRAX scanner and processed using caseviewer software. The antibodies used are CK8/18 abcam, ab53280 (1–1000 dilution), and Ki67 cst, 12202T (1–500 dilution).

For IF staining of wholemount organoids, organoids were fixed with 4% PFA on ice for 1 h, then blocked and permeabilized using 1% Triton X-100 and 1% FCS in PBS0 for 1 h at RT. Staining was performed in 0.3% Triton X-100 and 0.0.5% FCS in PBS overnight at 4° on gently shaking. Stained organoids were imaged using a Leica SP5 or SP8 confocal microscope. Images were processed using Leica software.

#### *Pten*/*Trp53* knock out in organoids

*Pten* and *Trp53* knock outs were generated by electroporation of Cas9-gRNA (RNP) complexes with the Lonza kit (Lonza, VVCA-1001), following instructions provided in the Lonza manual. Briefly, 1 million dissociated organoid cells were resuspended with nucleofection buffer, RNP complexes, and electroporation enhancer (IDT 1075915, 1:1molar ratio to cRNP) in a total volume of 100 μL. The cell suspension was transferred to a nucleofection cuvette and nucleofected using Lonza Amaxa Nucleofector II (program T-030). Cells were centrifuged at 400g for 5min and seeded for culture in matrigel, with organoid media. All guide RNAs used for the study were generated using the CRISPR Design Tool (http://crispr.mit.edu). Following gRNA sequences were used for the study:

*Pten* guide: ACCGCCAAATTTAACTGCAG.

*Trp53* guide: ACCCTGTCACCGAGACCCC.

non-targeting guide: CTTCACGCCTTGGACCGATA.

#### *Gata3* and *Pou2f1* KO in organoids

*Gata3* and *Pou2f1* gRNAs were cloned into lenti-lvt-pUSEG-GFP plasmid. Cas9 was transduced with lentiCas9-blast (addgene plasmid 52962). The organoids were infected with Cas9-blast and gRNA virus and selected with 2ug/ml of puromycin and 10ug/ml of blasticidin. After 5 days of selection, KO was confirmed by western blot assay on whole cell fractions. Guide 2 for Gata3 and guide 1 for *Pou2f1* were more efficient in knocking out their respective genes. These lines were subsequently used in multiomic assays. All guide RNAs used for the study were generated using the CRISPR Design Tool (http://crispr.mit.edu). Following gRNA sequences were used for the study:

*Gata3* guide 1: GGGACACGATCCTCAGCACA.

*Gata3* guide 2: GTTGCAGTTTCCTTGTGCTG.

*Pou2f1* guide 1: TCCCGTTCCTTCCTCTCCCG.

*Pou2f1* guide 2: TCGGGAGAGGAAGGAACGGG.

non-targeting guide: CTTCACGCCTTGGACCGATA.

#### Single particle tracking at fast framerates (fast SPT)

SPT experiments were conducted on a custom-built Nikon TI microscope with a 100x/NA 1.49 oil-immersion objective to allow for Highly-inclined Laminated optical (HiLo) illumination.^39^

About 18–24 h prior to imaging, LnCAP-AR cells were plated on MatTek #1.5 glass-bottom dishes by first coating the dishes in 0.01% poly-L-lysine and rinsing with distilled water. The day of imaging, cells were stained for ~5 min with 1 nM JFX650-HaloTag ligand and 10 nM JFX549-HaloTag ligand,^40^ then rinsed with pre-warmed media and incubated in more media for 20–30 min to allow for unbound dye to wash out. The cells were then immediately imaged, during which they were kept in an incubation chamber maintaining a measured 37°C 5% CO2 environment.

Cells were located using a 561 nm laser and imaged with the 635 nm laser in the following way for 10000 cycles: 7.48 ms frame interval (7 ms and a 0.48 ms camera transition time), 1 ms pulsed 635 nm laser powered to 1100 mW at the beginning of the camera integration phase, 0.48 ms 405 nm laser during the camera transition time to reactivate dyes that have entered a dark state. 13–22 cells of each clonal line were imaged each day and repeated over 3 days; one day’s results are shown, though the data are very reproducible day-to-day.

Spots from movies were detected, subpixel-localized, and connected by quot (https://github.com/alecheckert/quot), and analyzed using saspt (https://github.com/alecheckert/saspt,^26^; a published open-source method that infers proportions of diffusive states using Bayesian inference. For ease of comparison, we sum and highlight the inferred proportion of molecules diffusing below 0.1 μm2 s-1.

#### Fluorescence recovery after photo bleaching (FRAP)

FRAP was performed on clonal LnCAP-AR lines, as well as the parental LnCAP-AR line exogenously expressing H2B-HaloTag as a control. Cells were stained with 50 nM TMR-HaloTag ligand for 5 min, then rinsed with pre-warmed media and incubated in more media for 20–30 min to allow for unbound dye to wash out. The cells were then immediately imaged, during which they were kept in an incubation chamber maintaining a measured 37°C 5% CO2 environment.

Cells were imaged on a ZEISS LSM 900 in confocal mode using a 63x oil-immersion objective using the 561 nm laser for acquisition. A 2 μm diameter circle was bleached with both the 561 nm and 405 nm lasers after 20 frames of initial acquisition, and the cell was imaged for ~150 s afterward to track recovery. The bleach spot was drift-corrected after imaging; recovery of the spot was divided by the intensity of a frame-matched nuclear mask.

### Processing of single cell RNA sequencing

#### Read alignment of single nucleus RNA sequencing

Single-cell RNA sequencing data (scRNA-seq) was processed using 10x Cell Ranger ARC software, which includes alignment and minimal cell filtering for both scRNA-seq and scATAC-seq. This pipeline was customized to align sequencing reads to the Mus musculus genome (mm10) plus an additional sequence for the Foxa1 dsRed reporter.

#### Filtering expression matrix

The ARC filtered count matrix was examined with output plots to visualize cell and gene coverage distributions, and mitochondrial and ribosomal counts (Figure S1L). Based on these plots several quality metrics were used to remove poor quality cells (those with fewer than 500 transcripts) or putative multiplets (defined as cells containing greater than 100K transcripts). Cells with greater than 40% mitochondrial reads were filtered out from further consideration.

#### Expression matrix normalization and variance stabilization

To account for technical variation and normalize expression data the R package Seurat^41^ was used on the filtered integer gene count matrix. The sctransform method^42^ was used to perform normalization, variance stabilization, and feature selection. We regressed out cell cycle scores and mitochondrial percentage using vars.to.regress parameter of SCTransform function.

#### Dimensionality reduction and visualization

A nearest neighbor graph of cells was computed and used as input to the Leiden algorithm for cell clustering. Dimensionality reduction and uniform manifold and approximation projection (UMAP) and force directed layout using force atlas (FA) were applied to visualize cell clusters. A total of ten different cell clustering resolutions were evaluated and scored by silhouette score, a clustering metric. UMAPs were generated and used to hypothesize cell similarities and identify clusters for differential expression testing. Quality control, filtering, normalization and clustering of all cells were performed resulting in 20825 cells. Visualization of single nuclei on a UMAP from scRNA organoids depicted genotypically distinct cell transcriptomes.

### Differential genes and pathways

Differential expression testing was performed using the R package Seurat with presto.^43^ Genes were ranked using the average log foldchange and a false discovery rate adjusted *p*-value. Differential expression of L1, L2, and Basal signatures compared across 6 conditions ranked by AUC were plotted (Figure 1H) and can be found in Table S2. A GSEA analysis with fsGSEA^44^ utilized a list of ranked genes per comparison and the collection of molecular signatures database.

#### Single nucleus Assay for Transposase-Accessible Chromatin with high-throughput sequencing data (scATAC-seq)

Single-cell Assay for Transposase-Accessible Chromatin with high-throughput sequencing data (scATAC-seq) was analyzed using the ARC filtered count matrix.

Cell filtering, feature selection, and analyses were performed using ArchR software.^45^ We generated output plots to visualize distributions of cell counts within promoters, gene bodies, and transcription start sites (TSS). Quality metrics were used to remove poor quality cells including low TSS enrichment (<4) or low number of unique nuclear fragment (<1000) or putative multiplets (defined as cells containing greater than 1M reads).

#### Peak and motif identification

Peaks with scATAC-seq data were called using MACS2^46^ through the ArchR interface. Differentially accessible regions weredefined as those having significant difference with log2 fold change ≥0.5. For motif detection we utilized 2 databases, 1) CisBP and 2) the Non-redundant TF motif matches genome-wide (Vierstra2020). Motif matching within peaks was performed using MotifMatcher with defaults. The gene identity of each position weight matrix match was recorded so that we retained only those motifs whose corresponding gene was expressed. To identify motifs statistically significant between comparisons we calculated motif chromVAR^47^ scores for cell and performed Spearman correlation of those scores to the motif’s gene expression values. Those motifs having positive correlation with expression and significant chromVAR scores were kept for further analysis.

#### Read alignment and peaks atlas construction for chromatin immunoprecipitation by sequencing (ChIP-Seq) datasets

Alignments and peak files generation were performed with custom bash scripts that conformed to the ENCODE consortium ChIP analysis pipeline. To begin sequencing reads were aligned to the Mus musculus genome, mm10 using bowtie2 (–max_fragment_length 2000 –trim-qual 15). PCR and optical duplicate read were removed using Picard’s mark duplicates. We filtered out lower quality alignments using samtools (samtools view -F 1804 -bSq 30).

The de-duplicated, quality filtered alignment files were input to MACS2 for peak calling with a permissive *p*-value cutoff ≤0.2 to provide sufficient signal for Irreproducible Discovery Rate (IDR) analysis. ENCODE blacklisted regions were removed from the results and the output saved as narrow peak files. To standardize peak length the narrow peak entries were centered at peak summits and flanking regions were extended by 250 nucleotides. Next, we utilized Irreproducible Discovery Rate (IDR)for between replicate reproducibility of summits with a false discovery rate (FDR) ≤ 0.01. A final atlas of peaks was constructed by combining results from the above steps.

#### Differential ChIP-seq peaks and motif identification

A PCA of AR ChIP peak data confirmed replicate similarity, and luminal samples (H247Y, G275X, WT) grouped to one side while EV grouped with *Ar* KO. Differential peaks were determined by DESeq2.^48^ Briefly, the atlas of peaks were normalized using DESeq2’s median of ratios normalization using a model design: **~0 + condition** for a one versus others comparison. K-means was used to assess the number of peak clusters for each ChIP-seq set. ChIPseeker^49^ was then used to annotate genomic features of the peaks (Figures S3A and S3B).

Motif calling was performed within peaks by the Motif Occurrence Detection Suite (MOODS) library through the motifmatcher function in R. For motif identification we searched 2 databases, 1) CIS-BP and 2) the non-redundant TF motif matches genome-wide (Vierstra2020), containing mouse and human motifs. We considered only motifs having a *p*-value cutoff of 5e-05, within a discovery window of 7 nt. against the background frequency of Mus musculus genome nucleotides. To discover motifs differentially enriched we performed a binomial test of the number of significant motifs in one sample’s peaks vs. other samples (Figures S3C and S3E).

POU2F1 ChIP data was aligned to mm10 genome using bowtie2 (–local_preset ″–very-sensitive-local –no-mixed –no-discordant –phred33″) and filtered using samtools (samtools view -F 1804 -bSq 30). Duplicates were marked using picardtools. Replicate peaks were assessed using Irreproducible discovery rate (IDR) and retained if significant at IDR score ≥830. The atlas of peaks were normalized and compared between conditions using DESEQ2. POU2F1 peak read counts were compared in Foxa1 G275X (*n* = 2607) vs. Empty Vector (*n* = 4371) and assessed for significance. Peaks significantly upregulated either in Empty Vector or Foxa1 G275X (adjusted *p*-value <0.05 & log2FC > 0.5) were used for motif discovery performed with MOODs library. Only motifs having a *p*-value cutoff of 5e-05, within a discovery window of 7 nt were considered hits. Motif enrichments were calculated using binomial *Z* score comparing each genotype against the background set of peaks from the scATAC-seq peak atlas. Specifically, if *p* represents the probability that a peak in the background set contains an occurrence of the motif, then the binomial *Z* score for a cluster of size *N* with *C* peaks containing the motif is $\left(C-Np\right)/\sqrt{Np\left(1-p\right)}$. Although these Z-scores do not incorporate a correction for multiple hypotheses, in practice the top-ranked motifs have such strong enrichments they would still be highly significant after correction.

#### Milo analysis

We generated pseudo-replicates per sample from our single cell expression data and performed differential abundance testing using Milo.^8^ Neighborhood creation was performed using the following parameters makeNHoods(refinement_scheme = “graph”, prop = 0.1, k = k, d = 30, refined = TRUE, reduced_dims = “PCA”). A design matrix to compare each mutant to the Empty Vector control was created and used as input to makeContrasts(). Neighborhood tests were performed for all model contrasts, saved and plotted to show differential neighborhoods.

#### Gaussian mixture model

Single cell gene signature scores for each cell were input as a matrix to the function densityMClust() to perform point density estimation by a Gaussian finite mixture model for model assessment using the Mclust package.^50^ We then used the Mclust() function to fit the data to a 2-component Gaussian mixture model for luminal/basal classification. This demonstrated 2 distinct densities and allowed per cell luminal or basal probability assignment (Figures S1G and S1H). We also fit a 3-component Gaussian mixture model for basal, luminal1, and luminal2 probability assignment to each cell and performed dimensionality reduction using MclustDR() to reduce dimensionality with a set of linear combinations, ordered by importance as quantified by associated eigenvalues of the original features, similarly to PCA. (Figure S1I). A discrete cell classification was made by assignment to the class with greatest probability. We then calculated a heatmap showing the top differential genes per cell classification (basal, luminal1, luminal2) (Figure S1F).

#### Hotspot analysis

To identify genes that covary in cells in an informative manner and organize these genes into modules we performed Hotspot analysis.^15^ We used the single cell knn graph as input to hotspot to compute autocorrelations and find informative genes. We then selected highly significant genes according to Hotspot having an FDR <0.01 (*n* = 1850) and computed local correlations before grouping into modules. Gene modules were assessed using gprofiler2 and three query databases: gene ontology (GO), Kyoto Encyclopedia of genes (KEGG) and Reactome (REAC). The top pathways (within top 5) from GO were annotated on the heatmap.

#### AR regulated targets

We utilized multiome data output that jointly sequences chromatin accessibility using scATAC-seq and gene expression using scRNA-seq from the same cell. For each modality cells were combined to generate an ATAC and an Expression signal corresponding to each lineage specific state (L1, L2, Basal). We then used SCARlink to predict the expression data of each gene from chromatin accessibility data by defining the genomic region around the gene body with additional 250KB flanking regions extending the gene’s transcription start and transcription end sites. These regions were segmented into 500-nt tiles and ranked using SCARlink-derived Shapley scores to indicate the importance of each tile to the prediction. For each gene the Shapley scores were converted to z-scores and adjusted for gene length and used to rank putative enhancers (tiles).

Next, we gathered Ar ChIP-seq data overlapped with significantly differential scATAC-seq peaks per lineage specific state to assign ChIP-seq peaks to a specific state (Figure S3F). We then cross-referenced Ar ChIP-seq peaks, accessible AR motifs (ANDR_18 or ANDR_19) and SCARlink length adjusted z-scores to pinpoint Ar-regulated enhancers of TF genes. The overlap of these signals was converted to a score by calculating the dot product of the length adjusted *Z* score from SCARlink, the normalized peak counts from scATAC-seq and the ChIP normalized counts. Those loci that contained SCARlink enhancers overlapped with motifs and overlap with ChIP data were ranked by their score and plotted.

### QUANTIFICATION AND STATISTICAL ANALYSIS

All statistical analyses were performed with available code in R or using GraphPad Prism unless otherwise specified. Replicate numbers and statistical details are reported in figure legends. Details on statistical parameters used are presented within each relevant STAR Methods section and the key resources table.

## Supplementary Material

1

2

3

4

SUPPLEMENTAL INFORMATION

Supplemental information can be found online at https://doi.org/10.1016/j.celrep.2026.116950.

## Figures and Tables

**KEY RESOURCES TABLE:** 

REAGENT or RESOURCE	SOURCE	IDENTIFIER

Antibodies		

FOXA1	Sigma	SAB2100835-100UL
Vinculin	Cst	13901S
GATA3	Abcam	RRID: AB_2819013
POU2F1	Abcam	RRID: AB_301658
CK8/18	Abcam	RRID: AB_869901
Ki67	Cst	12202T
Cyclophillin B	Cst	RRID: AB_2799247
TBP	Abcam	RRID: AB_1281140

Bacterial and virus strains		

One shot Stbl3^™^ chemically competent E. coli	Thermofisher scientific	C737303

Chemicals, peptides, and recombinant proteins		

RPMI-1640	Thermofisher scientific	11875093
FBS	Thermofisher scientific	A5256701
Collagenase type II	Gibco	N/A
TrypLE	Gibco	N/A
DMEM	ATCC	30–2003
Lipofectamine 2000 reagent	Invitrogen	N/A
EGF	PeproTech	315–09
Y-27632	Selleck Chemicals	S1049
Mycoplasma test	Lonza	LT07-318

Deposited data		

Raw and processed data	This paper	GEO: GSE285574

Experimental models: Cell lines		

LNCaP-AR	Chen et al.^36^	Chen et al.^36^
Human HEK293	ATCC	CRL-1573

Experimental models: Organisms/strains		

Organoids	Adams et al.^1^	Adams et al.^1^

Oligonucleotides		

CGGGTCTGGaatacacacct[tgg]	This paper	WT
caagtgcgagaagcAGCCGG[GGG]	This paper	GX
ACCGCCAAATTTAACTGCAG	This paper	*Pten* guide
ACCCTGTCACCGAGACCCC	This paper	*Trp53* guide
CTTCACGCCTTGGACCGATA	This paper	non-targeting guide

Software and algorithms		

R	V4.4.2	https://cran.r-project.org;
Seurat	V5.3.1	https://satijalab.org/seurat;
ArchR	V1.0.2	https://www.archrproject.com/index.html
DESeq2	V1.44.0	https://bioconductor.org/packages/release/bioc/html/DESeq2.html
Cellranger-arc	V2.0.2	https://www.10xgenomics.com/support/software/cell-ranger-arc/latest
STAR	V2.7.2a	https://github.com/alexdobin/STAR
Scarlink	V1.0.0	https://github.com/snehamitra/SCARlink
Palantir	V1.4.0	https://github.com/dpeerlab/Palantir
MACS2	V2.2.9.1	https://pypi.org/project/MACS2/
IDR	V2.0.2	https://github.com/nboley/idr
Bowtie2	V2.5.1	https://bowtie-bio.sourceforge.net/index.shtml
Picardtools MarkDuplicates	V3.0.0	http://broadinstitute.github.io/picard/
Milo	V2.0.0	https://github.com/MarioniLab/miloR
Hotspot	V0.9.0	https://yoseflab.github.io/Hotspot/#
Deeptools	V3.5.6	https://deeptools.readthedocs.io/en/stable/
GraphPad Prism	10.6.1	https://www.graphpad.com/
Leica	LAS EZ	https://imillermicroscopes.com/pages/software-download
ImageJ	1.54g	https://imagej.net/ij/

Other		

Processed data	Li et al.^4^	https://pubmed.ncbi.nlm.nih.gov/32238934/

## Highlights

- Primary and metastatic prostate cancers harbor FOXA1 mutations at similar frequencies
- These FOXA1 alterations skew normal epithelial lineage specification
- Mutant FOXA1 reprograms cells by hijacking AR-FOXA1 regulatory networks
- An L1-like state is defined by a hybrid AR-FOXA1 motif and cooperation with POU2F1
