# Supplementary material for: FOXA1 mutations co-opt nascent transcription factor networks in partnership with androgen receptor to enhance prostate tumorigenicity
Source: Cell Rep. Author manuscript; Available in PMC 2026 Apr 5. (PMC13050545; doi:10.1016/j.celrep.2026.116950)
Supplement: 1 [file NIHMS2151731-supplement-1.pdf]

Cell Reports, Volume *45*

Supplemental information

FOXA1 mutations co-opt nascent transcription factor networks in partnership with androgen receptor to enhance prostate tumorigenicity

Erik M. Ladewig, Abbas Nazir, Tyler Park, Vinson B. Fan, Zhendong Cao, Jacob Hawk, Lauren Kelly, Robert Tjian, Christina S. Leslie, and Charles L. Sawyers

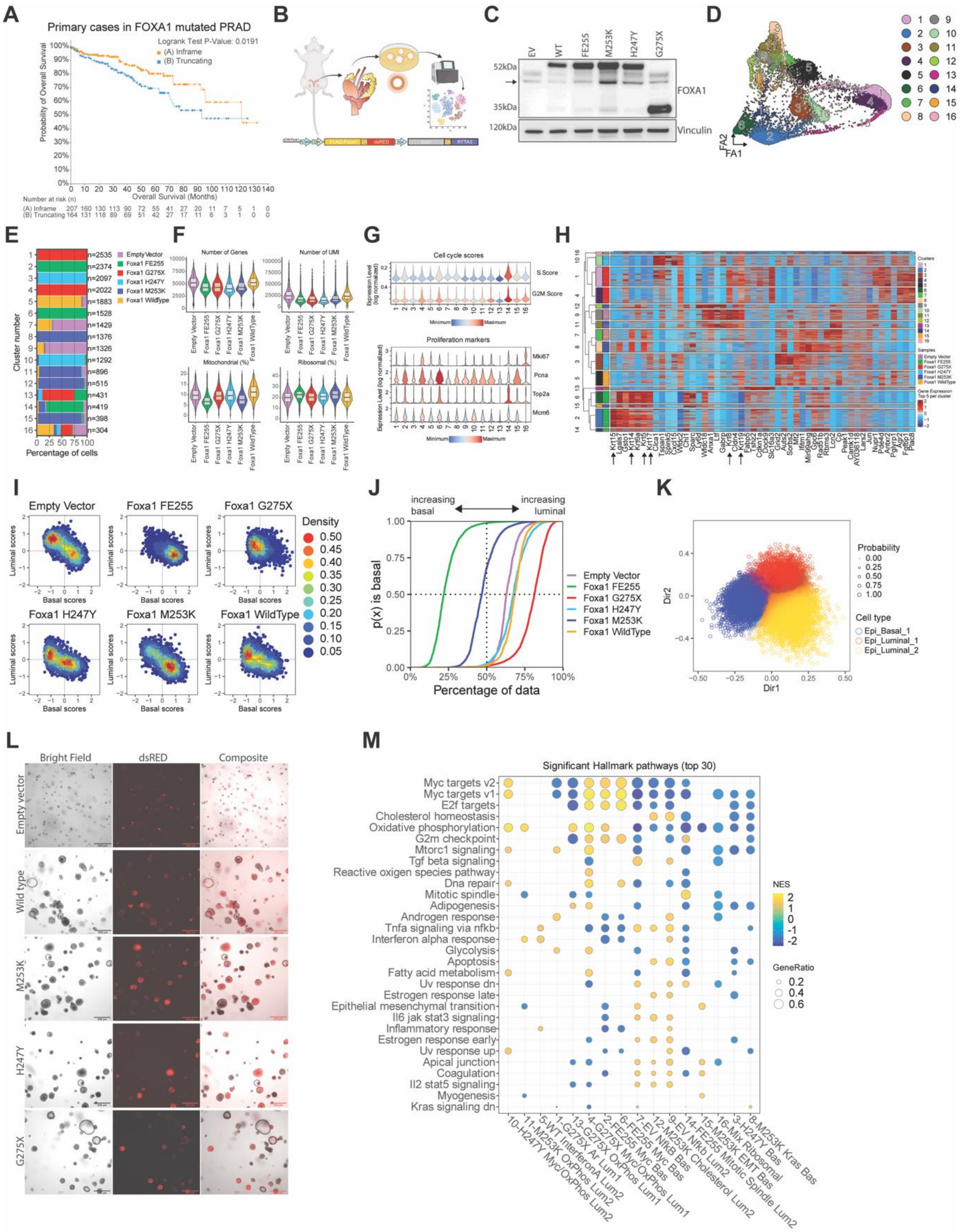
A
Primary cases in FOXA1 mutated PRAD
Logrank Test P-Value: 0.0191
(A) Inframe
(B) Truncating
Probability of Overall Survival
Overall Survival (Months)
Number at risk (n)
(A) Inframe 207 160 130 113 90 72 55 41 27 20 11 7 5 1 0
(B) Truncating 164 131 118 89 69 51 42 27 17 11 6 3 1 0 0
B
C
EV
WT
FE255
M253K
H247Y
G275X
52kDa
35kDa
120kDa
FOXA1
Vinculin
D
FA1
FA2
E
Cluster number
Percentage of cells
n=2535
n=2374
n=2097
n=2022
n=1883
n=1528
n=1429
n=1376
n=1326
n=1292
n=896
n=515
n=431
n=419
n=398
n=304
Empty Vector
Foxa1 FE255
Foxa1 G275X
Foxa1 H247Y
Foxa1 M253K
Foxa1 WildType
F
Number of Genes
Number of UMI
Mitochondrial (%)
Ribosomal (%)
G
Cell cycle scores
S.Score
G2M.Score
Proliferation markers
Mki67
Pcna
Top2a
Mcm6
Minimum
Maximum
H
Clusters
Samples
Gene Expression Top 5 per cluster
I
Empty Vector
Foxa1 FE255
Foxa1 G275X
Foxa1 H247Y
Foxa1 M253K
Foxa1 WildType
Luminal scores
Basal scores
Density
J
increasing basal
increasing luminal
p(x) is basal
Percentage of data
Empty Vector
Foxa1 FE255
Foxa1 G275X
Foxa1 H247Y
Foxa1 M253K
Foxa1 WildType
K
Probability
Cell type
Epi_Basal_1
Epi_Luminal_1
Epi_Luminal_2
Dir1
Dir2
L
Bright Field
dsRED
Composite
Empty vector
Wild type
M253K
H247Y
G275X
M
Significant Hallmark pathways (top 30)
Myc targets v2
Myc targets v1
E2f targets
Cholesterol homeostasis
Oxidative phosphorylation
G2m checkpoint
Mtorc1 signaling
Tgf beta signaling
Reactive oxigen species pathway
Dna repair
Mitotic spindle
Adipogenesis
Androgen response
Tnfa signaling via nfkb
Interferon alpha response
Glycolysis
Apoptosis
Fatty acid metabolism
Uv response dn
Estrogen response late
Epithelial mesenchymal transition
Il6 jak stat3 signaling
Inflammatory response
Estrogen response early
Uv response up
Apical junction
Coagulation
Il2 stat5 signaling
Myogenesis
Kras signaling dn
NES
GeneRatio
10-H247Y Myc/OxPhos Lum2
11-M253K OxPhos Lum2
5-WT InterferonA Lum2
1-G275X Ar Lum1
13-G275X Myc/OxPhos Lum1
4-G275X Myc/OxPhos Lum1
2-FE255 Myc Bas
6-FE255 Myc Bas
7-EV NfkB Bas
12-M253K Cholesterol Lum2
9-EV Nfkb Lum2
14-FE255 Mitotic Spindle Lum2
15-M253K EMT Bas
16-Mix Ribosomal
3-H247Y Bas
8-M253K Kras Bas

Fig S1. FOXA1 mutations frequently found in patient prostate tumors drive epithelial cell fate changes in prostate organoids

(**A**) Survival plot in primary prostate cases having In-Frame or Truncating FOXA1 mutation. (**B**) Experimental design of FOXA1 mutant organoids engineered from mouse prostate. (**C**) Western Blot of FOXA1 mutants used in study, all of which are Flag-tagged (see Methods). Arrow indicates endogenous FOXA1 protein. (**D**) scRNA-seq UMAP colored by Leiden clusters. (**E**) Bar plot of clusters defined by Leiden clustering of scRNA-seq data. Cluster number on left axis and number of cells annotated on right side. (**F**) Violin plots of scRNA-seq basic quality metrics used per sample. (**G**) Violin plots of Cell cycle scores (top) and proliferation markers (bottom) by cluster number. (**H**) Differential gene expression (DGE) heatmap by cell cluster showing prostate luminal (*Krt8*, *Krt19*) and basal (*Krt5*, *Krt14*, *Krt17*) cell markers among top DGE. (**I**) 2D density plots of luminal and basal scores per cell used as motivation for multivariate Gaussian fitting. (**J**) Empirical cumulative distribution function (ECDF) of basal cell probabilities (luminal prob. = 1-basal prob.) per genotype. Curves shifted to the left show higher probability of cells as basal and right shift show higher proportion of cells as luminal. (**K**) PCA-based dimensionality reduction after model-based clustering and classification. Points represent cells colored by epithelial cell type. (**L**) Organoid bright field staining of transgene in DSRed for EV, WT, M253K, H247Y, and G275X genotypes. (**M**) Hallmark pathways assessed via GSEA and shown per cell cluster.

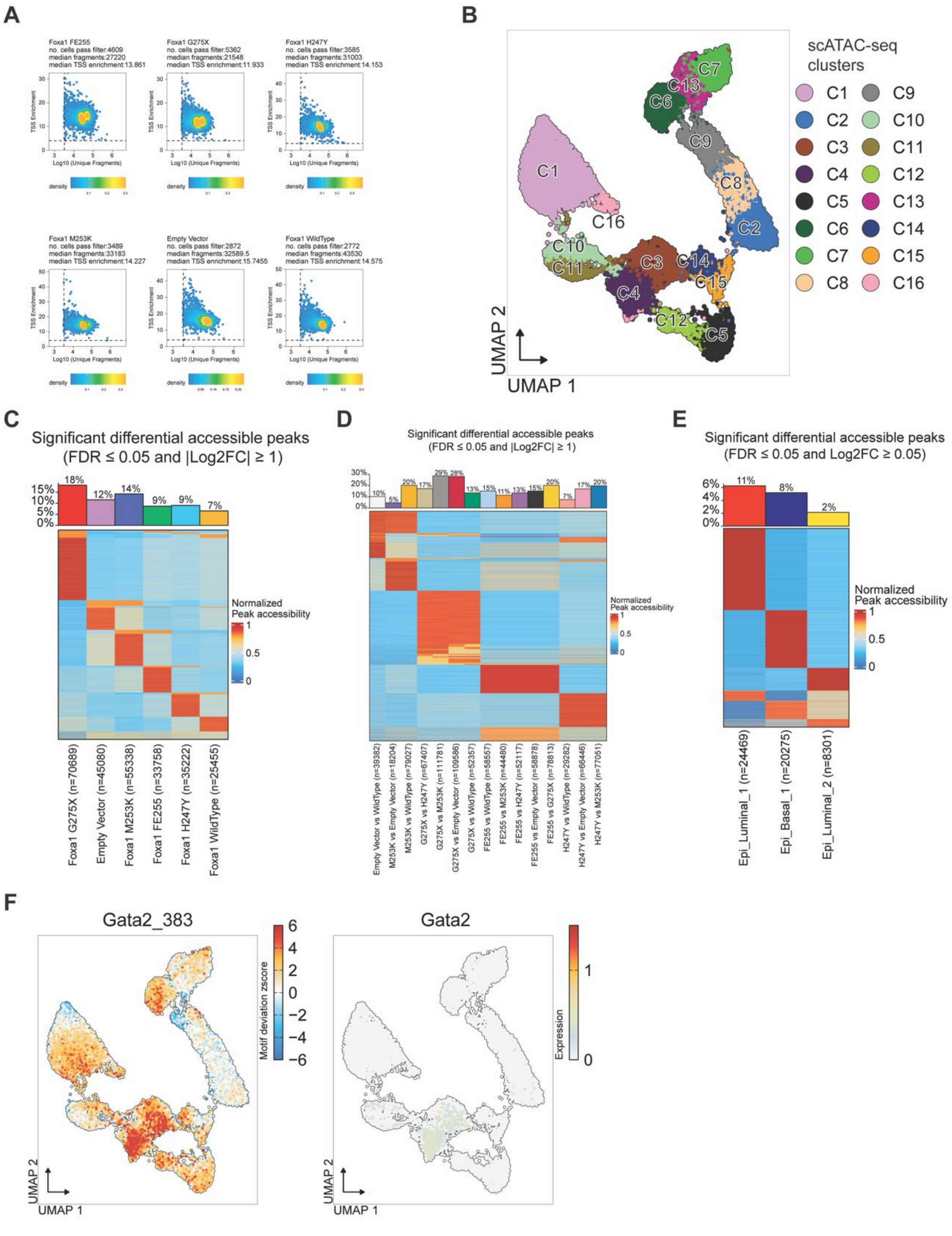

Fig S2. FOXA1 single nucleus chromatin accessibility reveals distinct transcription factor motifs that integrate luminal / basal expression programs

(**A**) scATAC-seq panels showing density of cells after quality filtering using Log10 (Unique fragments) on the x-axis and TSS Enrichment score (ratio of reads in TSS vs flanking regions) on the y-axis. (**B**) scATAC-seq UMAP of cells colored by leiden clustering. (**C**) Heatmap of average peak accessibilities per genotype that are significantly differential in one compared to all other genotypes (FDR ≤ 0.05 and $|Log2FC| \geq 1$). (**D**) Pairwise comparison heatmap of average peak accessibilities significantly differential (FDR ≤ 0.05 and $|Log2FC| \geq 1$). (**E**) Heatmap of average peak accessibilities per cell type that are significantly differential (FDR ≤ 0.05 and $|Log2FC| \geq 1$). (**F**) UMAPs of GATA2 chromatin accessibility motif deviation score (left) and expression of mRNA (right).

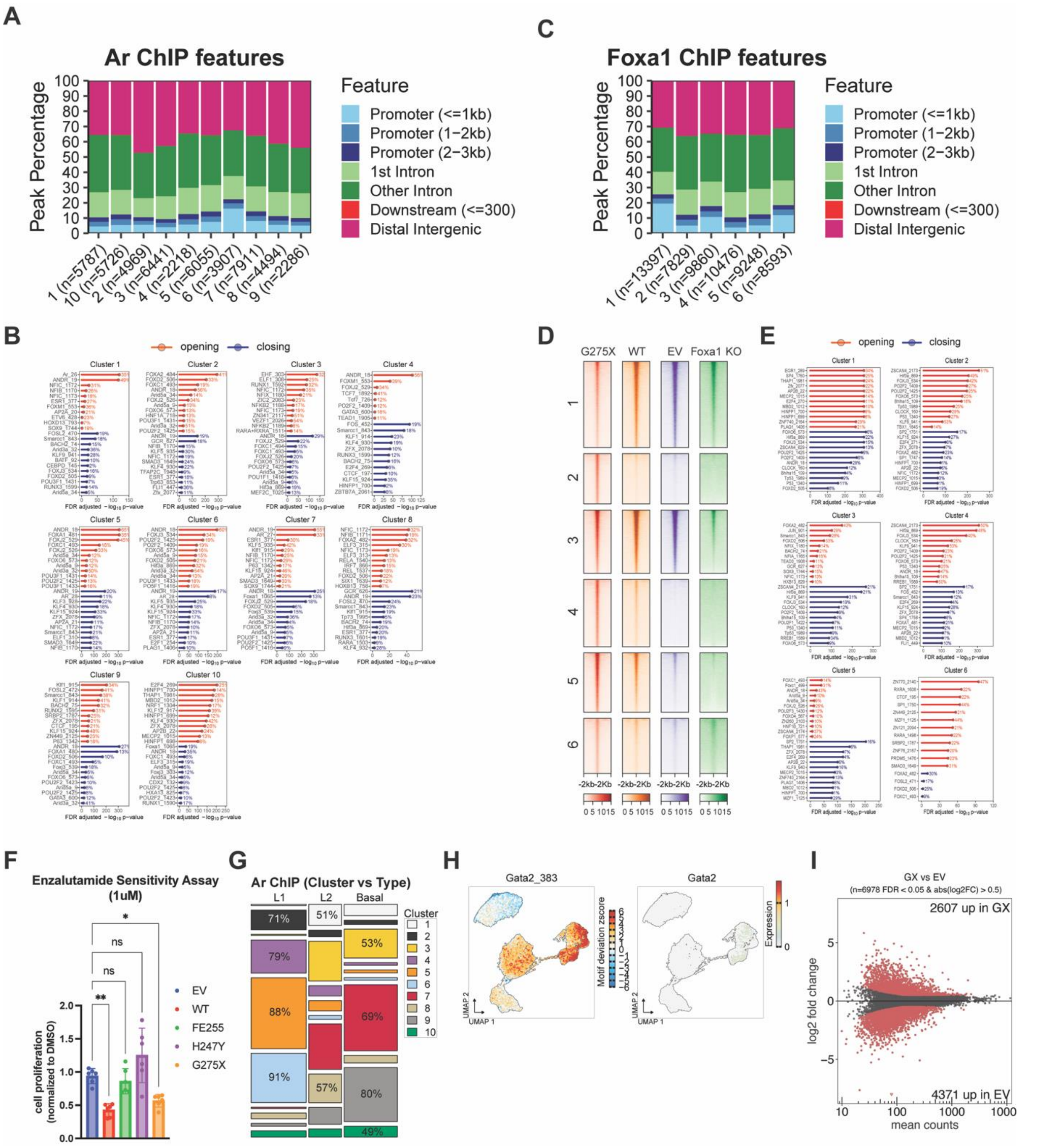

Fig S3. FOXA1 G275X cooperates with Androgen receptor by enriching for an alternative AR motif and reinforces AR target gene expression.

(**A**) AR ChIP-seq peaks labeled by % of features. (**B**) Top motifs detected within AR ChIP-seq peaks per cluster. (**C**) FOXA1 ChIP-seq peaks labeled by % of features. (**D**) Tornado plot of

FOXA1 ChIP-seq signal after K-means clustering in G275X, WT, EV, and *Ar* KO samples. (**E**) Top motifs detected within FOXA1 ChIP-seq peaks per cluster. (**F**) Organoid sensitivity measurements to 1 micromolar of the Androgen Receptor antagonist Enzalutamide. Cell proliferation measurements are shown normalized to the Empty Vector control. Pairwise statistical significance is indicated above braces. (**G**) Tile plot displaying AR ChIP-seq peaks clustered and categorized into epithelial cell types (basal, L1, L2) based on scATAC-seq overlap. Percentages representing the fraction of peaks by epithelial cell type are only sown for those significantly different between epithelial cell types. (**H**) UMAPs of GATA2 chromatin accessibility motif deviation score (left) and expression of mRNA (right). (**I**) MA plot of POU2F1 ChIP peaks. G275X significant peaks (FDR < 0.05 and |log2FC| > 0.5) are shown in red above the 0 on the yaxis. EV significant peaks (FDR < 0.05 and |log2FC| < 0.5) are shown in red with a -log2FC and below the 0 line on the y-axis.

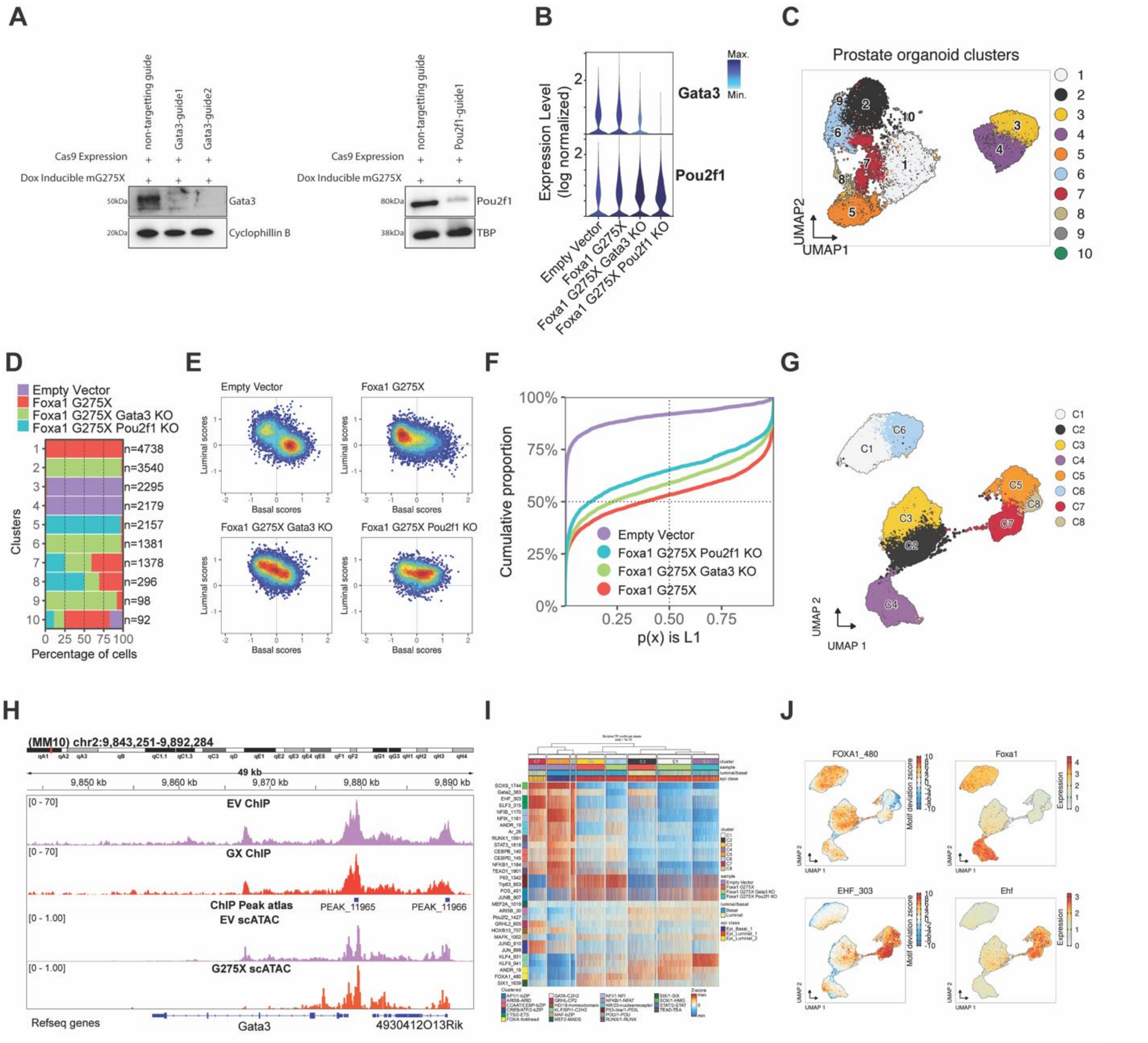

Fig S4. FOXA1 G275X enriches POU2F1 and GATA3 transcription factors to maintain luminal identity.

(**A**) GATA3 and POU2F1 expression with non-targeting or sgRNA guides. (**B**) Expression levels of *Gata3* and *Pou2f1* in genotypes showing decreased expression of Gata3 in both knockouts. (**C**) UMAP with cluster labels of scRNA-seq expression data. (**D**) Barplot showing percentage of genotype within clusters. (**E**) 2D scatter plots of assigned basal and luminal scores for each genotype's cells. Densities are calculated for each showing different enrichment per quadrant i.e. double positive, luminal, double negative, basal (I, II, III, IV). (**F**) ECDF shows probability of cell

to be L1 vs cumulative proportion of cells per genotype. G275X contains the greatest number of cells likely to be L1, second most likely is *Gata3* KO, third is *Pou2f1* KO, and EV with lowest probability of L1 cells. (**G**) scATAC-seq UMAP with cell clusters labeled. (**H**) Pou2f1 ChIP and snATAC-seq tracks for Empty Vector-EV (purple) and GX275-GX (red) show two Pou2f1 ChIP-seq peaks within 10KB upstream of the Gata3 locus (shown in IGV). (**I**) Heatmap of chromVAR motif accessibility z-scores hierarchically clustered. Motifs were selected based on high accessibility and correlated expression per cell. (**J**) Umaps of chromatin accessibility (left) and corresponding gene expression (right) for Foxa1 (top) and Ehf (bottom). *Foxa1* expression increased in G275X *Pou2f1* KO cluster although accessibility is like other G275X clusters. The ETS transcription factor Ehf (bottom) has increased accessibility and expression in the Empty Vector clusters.

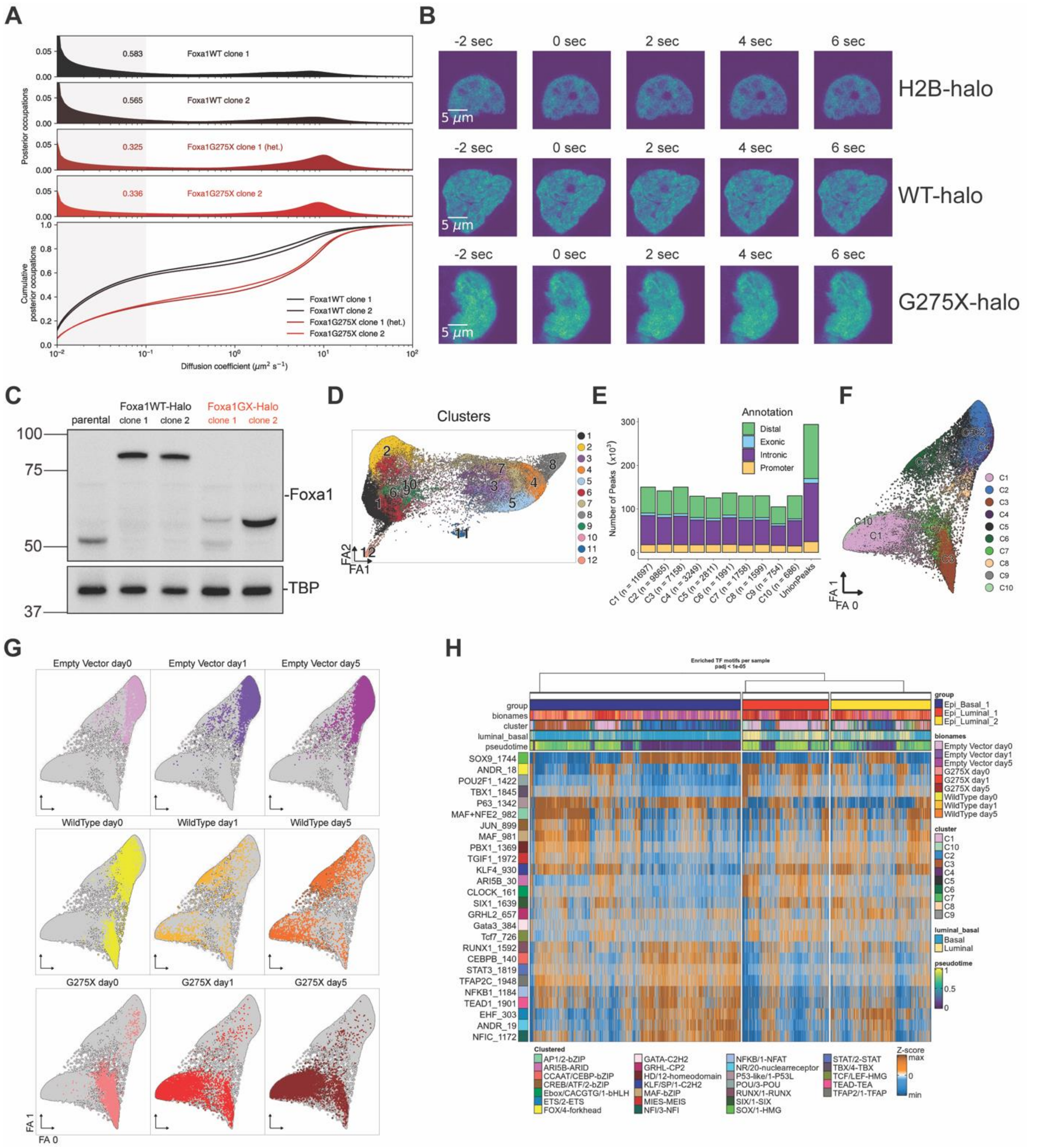

Fig S5. Faster activation of luminal identity in G275X mutant

(**A**) Experimental small particle tracking data (SPT) for WT and G275X with one replicate each.

(**B**) Fluorescence recovery after photobleaching (FRAP) was performed on clonal LnCAP-AR lines, as well as the parental LnCAP-AR line exogenously expressing H2B-HaloTag as a control.

(**C**) Western blot of parental, FOXA1 WT-halo and FOXA1 G275X-halo tagged proteins.

(**D**) scRNA-seq force directed layout (FA) colored by leiden clustering. (**E**) Number of peaks within scATAC-seq clusters annotated with peak categories. (**F**) scATAC-seq FA with cells colored by cluster. (**G**) scATAC-seq FA per genotype and day. (**H**) scATAC-seq heatmap clustered by genotype and day. Top motifs are labeled on the left with chromVAR z-scores shown per cell (columns).
